# IL-6-mediated tumorigenicity and antioxidant state in squamous cell carcinoma cells are driven by CD109 via stabilization of IL-6 receptor-alpha and activation of STAT3/NRF2 pathway

**DOI:** 10.1186/s40164-025-00630-x

**Published:** 2025-05-02

**Authors:** Amani Hassan, Tenzin Kungyal, Shufeng Zhou, Meryem Blati, Kenneth Finnson, Nick Bertos, Nahid Golabi, Nader Sadeghi, Sampath Loganathan, Anie Philip

**Affiliations:** 1 Divisions of Plastic Surgery, and Surgical and Interventional Sciences, Department of Surgery, McGill University, Montreal, QC Canada; 2 Divisions of Dermatology and Experimental Medicine, Department of Medicine, McGill University, Montreal, QC Canada; 3 Divisions of Thoracic and Upper GI Surgery, Department of Surgery, McGill University, Montreal, QC Canada; 4 Department of Otolaryngology, Head and Neck Surgery, and Division of Experimental Medicine, McGill University Health Center, Montreal, QC Canada; 5 Montreal General Hospital, 1650 Cedar Avenue, Room C10-148.4, Montreal, QC H3G 1A4 Canada

**Keywords:** Squamous cell  carcinoma, CD109, IL6Rα, STAT3, NRF2, Stemness, Antioxidant pathway

## Abstract

**Background:**

Squamous cell carcinoma (SCC) is a prevalent malignancy and there are limited options to block the recurrence and metastasis that often occur in SCC patients. Although IL-6, a proinflammatory cytokine, is strongly implicated in SCC pathogenesis, its mechanism of action is poorly understood. The GPI-anchored membrane protein CD109 is frequently overexpressed in SCC and is associated with malignant transformation. The current study aims to investigate whether CD109 interacts with IL-6 receptor alpha (IL6Rα) and promotes IL-6-mediated oncogenic signaling to drive SCC progression.

**Methods:**

IL6Rα interaction with CD109 was determined by coimmunoprecipitation, immunohistochemistry, immunofluorescence and FACS analysis using human SCC (oral and vulvar) cell lines and human oral SCC tumors versus control tissue. Regulation of IL-6-induced signaling and antioxidant responses by CD109 was analyzed via STAT3/NRF2/SOD1/HO1 pathway activation. Regulation of IL-6-mediated tumorigenicity by CD109 was determined using stem cell marker expression and a spheroid formation assay. Clinical validation was achieved using genomic and proteomic analysis of oral SCC tumors and of head and neck SCC patient data.

**Results:**

We show that CD109 interacts with and stabilizes IL6Rα expression and promotes IL-6/STAT3/NRF2 pathway in oral and vulvar SCC cells. Loss of CD109 attenuates this pathway leading to loss of cancer cell stemness and decreased expression of superoxide dismutase1 and heme oxygenase-1, antioxidant proteins important for cell survival after chemotherapy. Furthermore, clinical validation of these findings was achieved through multi-omic analysis of oral SCC tumors and of head and neck SCC patient data.

**Conclusions:**

This work uncovers a previously unidentified mechanism in which CD109 serves as an essential regulator of IL6Rα expression and IL-6 mediated signaling in SCC cells, promoting stemness and antioxidant state, mechanisms known to mediate therapy resistance in SCC. Our findings establish a mechanistic validation for investigating the therapeutic utility of the CD109/ IL6Rα/STAT3/NRF2 pathway in SCC.

**Graphical Abstract:**

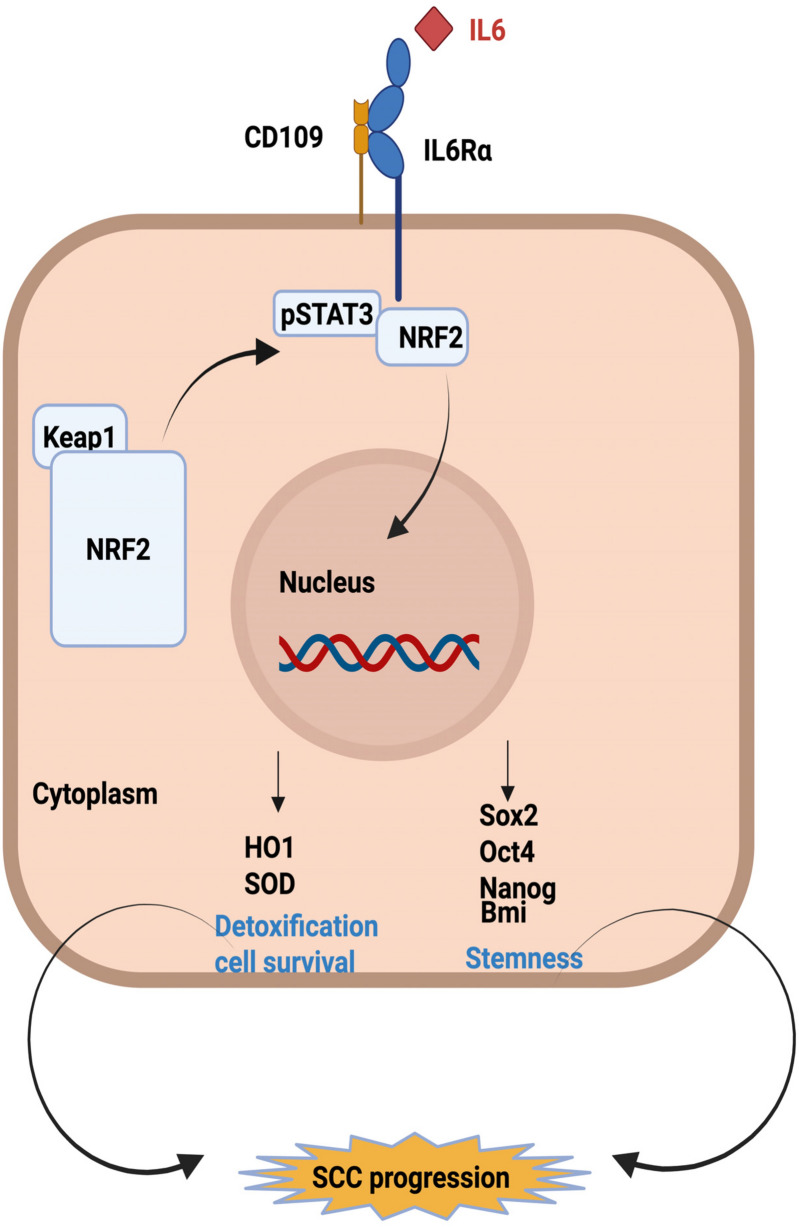

**Supplementary Information:**

The online version contains supplementary material available at 10.1186/s40164-025-00630-x.

## Introduction

Squamous cell carcinoma (SCC) is one of the most common solid cancers in humans and one of the leading causes of death worldwide [22]. Due to increased exposure to carcinogens such as ultraviolet radiation and human papillomavirus (HPV) infection, the incidence is increasing significantly [9, 42]. Despite intensive research, there has been limited success in blocking recurrence and metastasis that occurs in a substantial number of SCC patients. Surgery, laser therapy and radiation therapy remain the most widely used treatments for SCC [1, 38]. To successfully establish SCC prevention and treatment strategies, it is necessary to better understand the molecular events that occur in SCC tumors.

CD109 is a cell surface glycosylphosphatidylinositol (GPI)-linked glycoprotein and is a member of the α2 macroglobulin (α2M) /C3, C4, C5 complement family of thioester-containing proteins [11, 26, 45]. The functional significance of CD109 was first identified by our group by showing that CD109 is a transforming growth factor-β (TGF-β) co-receptor that negatively regulates TGF-β1 signaling [11] and inhibits TGF-β responses such as extracellular matrix synthesis and fibrosis [2, 27, 53, 54, 56]. CD109 has recently emerged as a major gene dysregulated in SCC tumors. CD109 expression is increased in SSC tumors from multiple tissue origins including skin (cSCC), oral cavity (oSCC), lung, vulva and bladder [6–8, 14, 15, 33, 39, 41, 43, 44, 59]. In addition, premalignant lesions of SCC that highly express CD109 were shown to have a greater risk of metastatic progression [6], suggesting that CD109 may play a causal role in the SCC progression. In this regard, CD109 KO mice have been shown to display decreased skin cancer progression in a chemical skin carcinogenesis model, as compared to wild-type littermates [48]. Delineating the functional significance of CD109 dysregulation in SCC will involve defining the molecular mechanisms by which CD109 may regulate SCC tumor progression. Recent reports by us [62] and others [32] suggest that CD109 promotes cancer progression by potentiating epidermal growth factor receptor (EGFR) signaling in SCC cells. CD109-mediated augmentation of EGF signaling has also been reported in lung adenocarcinoma and in glioblastoma cells [25, 60]. Identifying the specific signaling pathways regulated by CD109 and how CD109 may regulate the balance of signaling via these pathways will provide critical insights into the precise mechanisms of CD109 action in SCC progression.

IL-6 is a proinflammatory cytokine whose excessive signaling is believed to play a key role in cancer progression [13]. The IL-6 receptor is composed of two subunits, IL6Rα and gp130, which are important for ligand binding and signal transduction respectively. The binding of IL-6 to its receptor leads to intracellular Janus kinase (JAK) phosphorylation and nuclear translocation of STAT3, leading to the regulation of gene transcription [18]. IL-6 can also activate other signaling pathways, including MAPK and Akt, which can elicit a variety of cellular responses [4, 57]. Over the past two decades, studies focusing on the association of IL-6 with clinicopathological features and oncological outcomes in SCC, have shown that IL-6 is strongly implicated in the pathogenesis of SCC [5]. The expression of IL-6 mRNA and protein was directly correlated with advanced tumor stage [63] and its elevated expression was associated with increased recurrence and decreased survival rate [52]. IL-6 serum concentrations were found to increase as tumor stage progressed [52]. IL-6 serum levels are reduced after treatment in SCC patients, and elevated IL-6 levels have been suggested to mediate chemo and radio-resistance in SCC [17, 35, 36, 46].

The mechanisms by which IL-6 mediates cancer progression and resistance to therapy are poorly defined. IL-6 signaling through JAK/STAT3 leads to epithelial-mesenchymal transition (EMT) in oral SCC [58] and stemness of gliomas [28] which may contribute to tumor progression. Also, IL-6 has been reported to play an important role in the induction of the immunosuppressive tumor microenvironment [50]. Furthermore, recent evidence indicates that IL-6 mediates resistance to therapy in oral SCC via the NRF2 (NF-E2-related factor 2) anti-oxidant pathway [29], a pathway recognized as one of the main cellular defense mechanisms against oxidative stress [49]. Under basal conditions, the NRF2 protein is sequestered by the repressor protein, Kelch-like ECH-associated protein 1 (KEAP1) in the cytoplasm and is constitutively degraded via the ubiquitin-proteosome pathway [23]. Under oxidative stress, NRF2 undergoes nuclear translocation by escaping KEAP1 repression and induces the transcription of cytoprotective genes [31]. Emerging evidence indicates that the NRF2 anti-oxidant pathway mediates resistance to therapy in cancer cells [16, 19, 34, 37, 40, 55] and that IL-6 promotes therapy resistance via NRF2 in SCC [29, 34].

Given the tumor-promoting role of CD109 and of IL-6 in SCC and the recent findings that CD109 potentiates JAK/STAT3 signaling in lung adenocarcinoma and glioblastoma cells, it was important to establish whether CD109 regulates IL6Rα signaling in SCC and determine the mechanisms involved. In the current study, we demonstrate that CD109 interacts with and stabilizes IL6Rα to promote the activation of the IL6Rα/STAT3/NRF2 pathway in SCC cells. Our findings establish CD109 as an essential regulator of IL-6-mediated oncogenic activity promoting cancer cell stemness and antioxidant state in SCC cells, providing mechanistic insights into IL-6-mediated cell survival and resistance to therapy in SCC.

## Materials and methods

### Reagents

For IL-6 treatments, cells were serum-starved for 24 h and then either left untreated or treated with 20 ng/ml IL-6 (PeproTech, #200-06) for 25 min to measure p-STAT3. To assess IL-6’s impact on stemness, proliferation, and EMT marker expression, cells were left untreated or treated with 50 ng/ml IL-6 for 48 h. Co-Immunoprecipitation (Co-IP) experiments were conducted by treating cells with 20 ng/ml IL-6 for 25 min. Protein synthesis was blocked using 0.5 mg/ml cycloheximide (Sigma; #C4859-1ML) for varying time points. STAT3 activation was inhibited with Stattic (Sigma; #573099-25MG), a potent STAT3 inhibitor, at 20 µM for 1 h. NRF2 activity was blocked with ML385 (Sigma; #SML1833-5MG) at 5 µM. Tocilizumab, a humanized monoclonal antibody against IL-6R, was used at 10 µg/ml for 24 h. Human IgG1 kappa isotype control (Genetex; #GTX16193) was employed as a control at the same concentration.

### Cell culture

The human vulvar squamous cell carcinoma A431 cell line (RRID: CVCL_0037) was cultured in Dulbecco's Modified Eagle’s Medium (DMEM; Gibco Life Technologies; #11995-065) supplemented with 10% fetal bovine serum (FBS, Gibco Life Technologies; #12483-020). Human tongue squamous cell carcinoma SCC9 cell line (ATCC CRL-9) was cultured in a 1:1 mixture of Dulbecco’s Modified Eagle’s Medium and Ham's F12 Medium containing 1.2 g/l sodium bicarbonate, 2.5 mM l-glutamine, 15 mM HEPES, and 0.5 mM sodium pyruvate supplemented with 400 ng/ml hydrocortisone and 10% FBS. For the protein degradation assay, we utilized CD109 overexpressing A431 and empty vector (EV) containing stable cell lines, cultured in DMEM supplemented with 10% FBS, geneticin, and penicillin–streptomycin. All cells were maintained in a humidified culture chamber at 37 °C with 5% CO_2_.

ATCC and LabCorp authenticated cell Lines used in this study.

### siRNA transfection

siRNAs targeting IL6Rα (Invitrogen; #AM16708-106148) with the sense sequence 5ʹGGCAUACCUUAAACAAGCUTT3ʹ and antisense sequence: 5ʹGAUGCAUGCUUGUCUUGCCTT3ʹ, GP130 (Invitrogen; # AM51331/106709) with the sense sequence: 5ʹGGCAUACCUUAAACAAGCUTT3ʹ and antisense sequence: 5ʹAGCUUGUUUAAGGUAUGCCTT3ʹ, STAT3 (Invitrogen; #S744/4390824) with the sense sequence 5ʹGGCUGGACAAUAUCAUUGATT3ʹ and antisense sequence 5ʹGAUCUAUCCAAAAUCAAGATT3ʹ, or NRF2 (Invitrogen; #S9491-4392420) with the sense sequence 5ʹGAAUGGUCCUAAAACACCAtt3ʹ and antisense sequence 5’UGGUGUUUUAGGACCAUUCTG3’ were transfected into parental WT A431 or SCC9 cells using Lipofectamine RNAiMAX Transfection Reagent (Invitrogen; #13778030) per the manufacturer’s instructions. Cells were serum-starved for 24 h at 72 h post-transfection.

### Immunofluorescence staining

Cells were fixed in 4% paraformaldehyde for 15 min, permeabilized in PBS/0.3% Triton X-100 (PBT) for 15 min, and blocked in 2% BSA in PBT for 1 h. For oral SCC patient tissues, the samples were deparaffinized in Xylene, followed by rehydration in Ethanol and water, and antigen retrieval was performed using Citrate buffer (Abcam; #ab93678 ). Primary antibodies (anti-CD109, anti-IL6Rα, anti-pSTAT3 (705), anti-STAT3) were added at 1:250 dilution in 2% BSA/PBT and incubated overnight at 4 °C. After washing with PBS, cells were labeled for 1 h with fluorophore-conjugated secondary antibodies (1:500 dilution) and mounted using prolong gold anti-fade reagent with DAPI. Images were captured using an Olympus microscope IX71.

### Western blot analysis

Cell lysates were prepared using Pierce RIPA buffer (ThermoFisher Scientific; #89900 ) supplemented with 1 × protease inhibitor cocktail (Roche; #04693116001 ), followed by centrifugation. The supernatant was collected for protein quantification via the Bradford Assay (Bio-Rad; #500–0006). Twenty micrograms of total protein per sample underwent Western blot analysis. Samples were resolved on a 10% polyacrylamide gel and transferred to nitrocellulose membranes (Bio-Rad; #9004700). Ponceau S staining (Millipore Sigma; #P3504) detected total protein, which was followed by blocking with 5% BSA in TBST and incubation with specific primary antibodies from Santa Cruz overnight at 4 °C: anti-CD109 (#sc271085), anti-STAT3 (#sc-8019), anti-phospho-STAT3 (Tyr 705) (#sc-8059), anti-IL6Rα (#sc-373708), anti-Sox2 (#sc-365823), anti-Oct4 (#sc-5279), anti-Nanog (#sc-374103), anti-Bmi (#sc-390443), anti-NRF2 (#sc-365949), anti-phospho-NRF2 (ser 40) (#ab76026, Abcam), anti-SOD1 (#sc-17767), anti-HO1 (#sc-136960) and anti-β-actin (#sc-47778).

Membranes were then probed with anti-rabbit IgG or anti-mouse IgG secondary antibodies (Cell Signaling Technology; #7074S, #7076S) diluted at 1:1000 for 1 h at room temperature. Protein bands were visualized using Clarity Max Western ECL Substrate (Bio-Rad; #1705060) and developed with an Omega LumTM C machine from Aplegen.

### Coimmunoprecipitation

For protein extraction, cells were lysed in Pierce IP Lysis Buffer (ThermoFisher Scientific; #87787) ). Immunoprecipitation was performed by incubating 500 μg of proteins with 2.5 μg/ml of Santacruz antibodies: anti-CD109 (#sc-271085), anti-IL6Rα (#sc-373708), anti-STAT3 (#sc-8019), or anti-NRF2 (# sc-365949) antibodies overnight at 4 °C. SureBeads™ Protein G Magnetic Beads ( Bio-Rad; #161-4023) were washed with PBS, blocked with 2% BSA/PBS overnight at 4 °C, then incubated with protein samples. Proteins were eluted using 1 × Laemmli buffer and ran alongside dual plus molecular weight ladders (Bio-Rad; #161-03745) in SDS-PAGE for approximately 90 min. Membranes were blocked and probed with the following antibodies: mouse monoclonal anti-CD109 (#sc271085), anti-IL6Rα (#sc-373708), anti-NRF2 (#sc-365949), anti-STAT3 (#sc-8019), or anti-CD130/gp130 Antibody (#sc-376280).

### Generation of knockout cell line with CRISPR/Cas9

The generation of A431 WT and KO is previously described by Zhu et al.[61]. For A431 WT and KO generation, the gRNA sequence (5′-CACCGACTATTGGGGTGGAGCTTC-3′) targeting exon 2 of CD109 was cloned into the pX458 CRISPR/Cas9-GFP vector (Addgene, Watertown, MA, USA). A431 cells were transfected with pX458/gRNA using lipofectamine 2000, sorted as single cells per well, and single CD109 negative cells were used to generate CD109 KO single-cell clones. Control A431 cells underwent the same cloning and GFP sorting procedure but retained CD109 expression and epithelial traits, referred to as A431 WT control cells. These control cells exhibited similar characteristics to parental A431 cells, as confirmed in previous studies (Figure S5A).

SCC9 WT and CRISPR/Cas9-mediated CD109 KO pools were obtained from Synthego Corporation (Redwood City, CA) (Synthego SO# 3036353-1). The gRNA sequence targeting exon 2 of CD109 is CTTCCAGGACAGAGACAGTG. Sequencing of WT and CD109 KO pools was performed using the primers: Forward: 5ʹTGCCAAATAGAGCAGTGGGC3ʹ and Reverse: 5ʹTGGGGTTTCTCTGCTATACTACC3ʹ. The KO pool exhibits a T nucleotide deletion in exon 2 (Figure S5B).

### ROS detection using the ROS-Glo™ H2O2 assay

ROS levels were measured using ROS-Glo™ H2O2 Assay kit from Promega (Promega; # G8820) according to manufacturer’s instructions. A431WT, SCC9 WT and their corresponding CD109 KO cells were seeded into white-walled, clear-bottom 96-well plates at a density of 10,000 cells per well in 80 µl of complete medium. Cells were allowed to adhere for 24 h and were then serum-starved for 24 h followed by treatment with 50 ng/ml IL-6 for 2 h, 4 h or 24 h at 37 °C.

After the treatment period, 20 µl of the H₂O₂ Substrate Solution from the ROS-Glo™ H2O2 Assay kit was added into each well. The plate was gently mixed and incubated for 6 h at 37 °C. Subsequently, 100 µl of the ROS-Glo™ Detection Solution was added to each well, and the plate was incubated at room temperature for 20 min in the dark. Luminescence was recorded using a microplate reader, with an integration time of 0.5–1 s per well.

### Flow cytometry analysis of cell surface expression

To measure cell surface expression of CD109 and IL6Rα, CD109 overexpressing A431 cells and empty vector (EV) containing A431 cells were serum starved for 24 h and then treated with 20 ng/ml IL-6 for 15 min or 1 h and then cells were detached, harvested and washed with cold PBS. Cells were then resuspended in staining buffer (2% FBS, 1 mM EDTA, PBS), blocked with Fc blocker (Invitrogen; #14-9161-71) to block Fc-mediated non-specific binding, and then stained with CD109-PE (ThermoFisher Scientific; Clone HU17, eBioscience™ ) and IL6Rα (Santa Cruz Biotechnology; #sc-373708) antibodies. Then cells were incubated with Alexa Flour 488 (ThermoFisher Scientific; #A11001) to detect the anti-IL6Rα antibody and then analyzed using FACSAria III.

### Tumor spheroid formation assay

Single cell suspensions (10,000 cells) of A431 or SCC9 WT and their corresponding CD109 KO or A431 empty vector (EVA431) or A431 overexpressing CD109 (OECD109 A431) cells were suspended in six-well ultra-low attachment plate (VWR; #29443-030) in DMEM/F12 Medium (Gibco; #11320033) with 20 ng/ml recombinant human fibroblast growth factor10 (FGF-10) (PeproTech; #100-26), 20 ng/ml hFGF2 (PeproTech; #100-18B), 9.5 µg/ml insulin (Sigma-Aldrich; # I9278) and 2% Gibco B-27 Supplement (ThermoFisher Scientific; #17504-044) at 37 °C in 5% CO_2_. The medium was changed once a week. After 2 weeks, individual spheroids were counted under an inverted microscope at 20× magnification. The percentage of cells capable of forming spheroids was calculated as follows: [(number of spheroids formed/number of cells plated) × 100].

### Immunohistochemistry (IHC) analysis

IHC was conducted on human tumor and adjacent control tissue obtained during surgery. Primary antibodies included mouse monoclonal anti-CD109 (Santa Cruz Biotechnology; #sc271085), IL6Rα antibody (ABclonal Technology; #A1570), NRF2 antibody ( Santa Cruz Biotechnology; #sc-365949), pSTAT3 antibody (Santa Cruz Biotechnology; #sc-8059), and STAT3 antibody (Santa Cruz Biotechnology; #sc-8019). Incubations were overnight at 4 °C. Secondary antibodies were used with the Mouse and Rabbit Specific HRP/DAB IHC Detection Kit (Abcam; #ab236466). Negative controls involved replacing the primary antibody with normal serum and incubating slides with PBS. IHC was performed on tissue from at least 3 patients.

### Collection of tumor and adjacent normal tissue samples from Oral HNSCC patients

The oral SCC samples were collected by Dr. N. Sadeghi and his team at the Department of Otorhinolaryngology-Head and Neck Surgery, McGill University Health Centre. The study was approved by the Research Ethics Committee at the McGill University Health Centre (MP-37-2019-4659) and conducted following approved guidelines. Patients provided written informed consent, and all experimental protocols were approved by Canada's Research Review Office (RRO).

Eligible patients were previously untreated, HPV negative, without a second primary tumor and seeking treatment at the same institution. Stringent inclusion and exclusion criteria were applied to define a precise study cohort. Eligibility required a confirmed diagnosis of oral cancer, an age range of 18 to 75, and no prior cancer treatment. Conversely, exclusion criteria included patients with nodal metastatic disease, severe comorbidities, or those who were pregnant or breastfeeding. Attrition rates were meticulously tracked, with efforts made to reduce dropout through patient support and consistent follow-up. The study considered sex as a biological variable, ensuring an equitable representation of both male and female participants to account for potential differences in disease progression and treatment outcomes. Comprehensive subject demographics were recorded, including age, race, socioeconomic status, and lifestyle factors like smoking and alcohol consumption. Blinding was used where possible, especially in outcome assessments, to minimize bias. A power analysis was performed during the study design to confirm that the sample size was adequate to detect significant differences. Clinical Data (including age and sex) of oral SCC patients who consented and generously provided the samples used for immunohistochemical analysis are summarized in Supplementary Table 1.

### Proteomic analysis

Data on head and neck squamous cell carcinoma (HNSCC) patients (n = 109) were extracted from the LinkedOmics database http://www.linkedomics.org. Patients were divided into CD109 high versus CD109 low groups using the median as a cutoff, and the analysis was performed using the top 25% of CD109 high (n = 27) versus the bottom 25% of CD109 low n = 27). The Pearson correlation between CD109 and NRF2 protein levels in patient groups were obtained using Graphpad Prism (RRID: SCR_002798).

Gene set enrichment analysis (GSEA) was performed using the same proteomic data (https://linkedomics.org/data_download/CPTAC-HNSCC/) (RRID: SCR_003199) which involved 9666 identified proteins. Patients were divided into CD109 high (n = 55) versus CD109 low (n = 54) groups using the median as a cutoff. GSEA was performed against the molecular signatures database (MSigDB) to focus on oncogenic gene sets (c6.all.v2022.1.hs.symbol.gmt) based on the reported data on NRF2 alterations in cancer, available from the WikiPathway (WP_NRF2_PATHWAY or M39454) and Broad Institute database[47]. The GSEA analysis represents enrichment gene sets with a strict False Discovery Rate (FDR) of less than 25% which was validated using 1000 random permutations.

### Gene expression analysis

Gene expression patterns were analyzed using The Cancer Genome Atlas (TCGA-Pan Cancer Atlas) head and neck squamous cell carcinoma datasets from cBioPortal for Cancer Genomics (https://www.cbioportal.org/). The dataset comprised of tumor samples from 523 patients. Correlation plots for the mRNA expression of CD109 versus that of IL6Rα or NRF2 target genes (AKR1B10, AKR1C1, AKR1C2, AKR1C3, GCLC, GCLM, NTRK2, SLC7A11, SRXN1, TXNRD1 and UCHL1) were generated, displaying p-values, Pearson and Spearman correlations, and regression lines.

### Survival analysis

The correlation of CD109 expression with disease-free survival and overall survival of HNSCC patients was done using the data of the same 523 HNSCC patients from the TCGA database by first stratifying the patients as CD109 high/IL6Rα high (n = 24 patients) versus CD109 low/IL6Rα low (n = 361patients). The survival probability was analyzed by a log-rank (Mantel-Cox) statistical test.

### Statistical analysis

All quantitative data are presented as mean ± SEM. Data was analyzed by two-tailed Student’s *t*-test or one-way and two-way Analysis of Variance (ANOVA). A *p*-value < 0.05 was considered significant.

## Results

### CD109 is a critical regulator of IL6Rα expression, potentiating IL-6-induced STAT3 activation in SCC cells

**Fig. 1:**
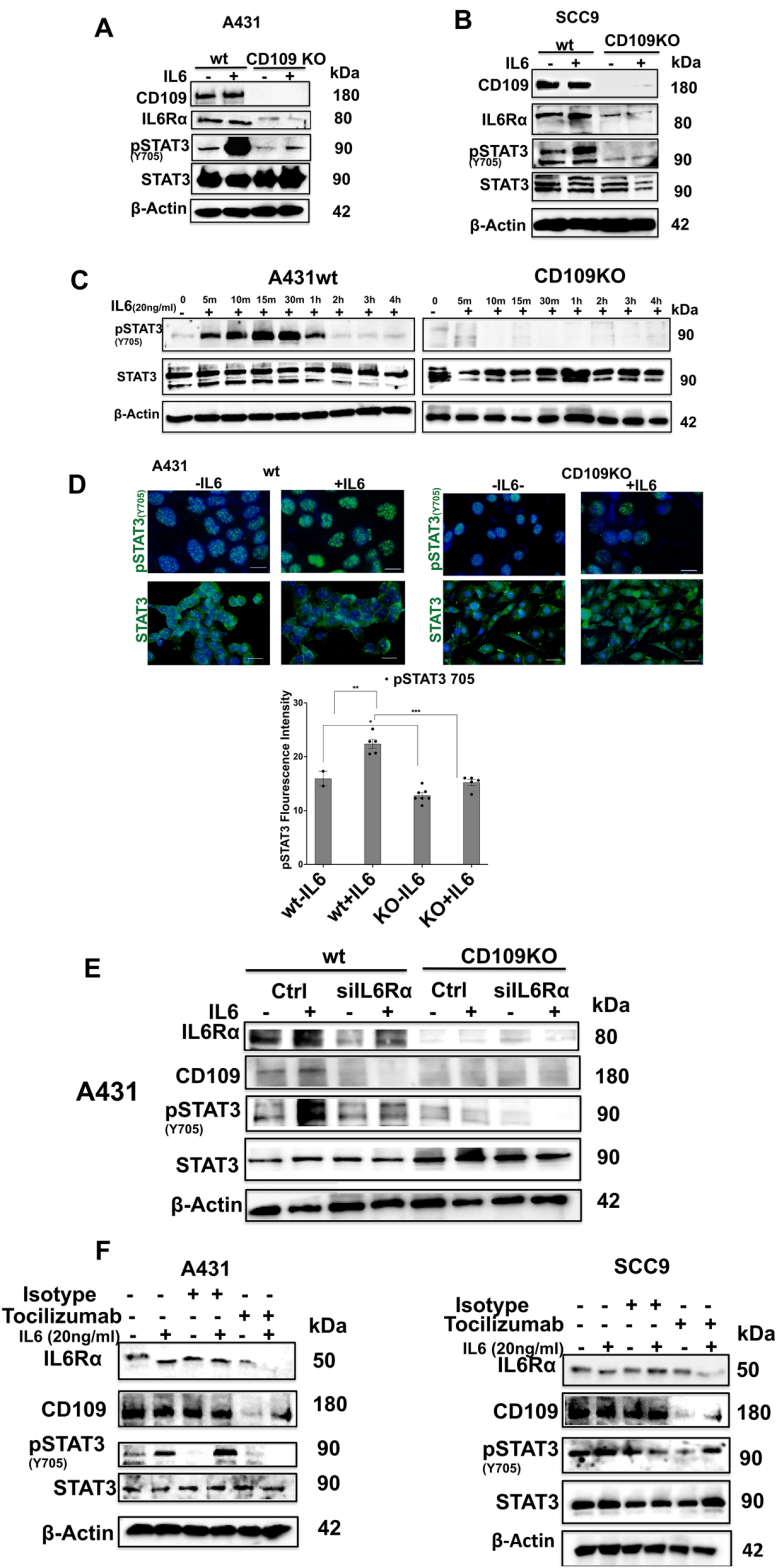
CD109 promotes IL6Rα stability and IL-6 induced phosphorylation of STAT3 in SCC cells. **A**, **B** A431, SCC9 wild-type (WT) cells and CD109 knockout (KO) cells were treated with IL-6 (20 ng/mL) for 30 min and the expression of CD109, IL6Rα and phosphorylation of the STAT3 (Y705) proteins were analyzed by Western blotting. Densitometric analysis of the data depicted in A and B are shown in supplementary data. **C** A431-WT and A431-CD109 KO cells were treated with IL-6 (20 ng/ml) for various time periods (0–4 h), and the levels of phospho-STAT3 (Y705) and total STAT3 were assessed by Western blot. β-Actin was used as a loading control. **D** Fluorescence microscopy showing the levels of phospho-STAT3 (Y705) (upper panels, green) and total STAT3 (lower panels, green) in A431-WT and A431-CD109 KO cells treated with IL-6 (20 ng/ml). Scale bar: 25 μm. **E** A431-WT and A431-CD109 KO cells transfected with IL6Rα siRNA or control siRNA and treated with IL-6 (20 ng/ml), and the expression of IL6Rα, CD109 and the levels of phospho-STAT3 (Y705) were analyzed by Western blotting. **F** A431 and SCC9 cells were treated without or with (20 ug) tocilizumab for 24 h and the expression of IL6Rα, CD109 and phosphorylation of STAT-3 (Y705) were analyzed by Western blotting. All results (**A**–**F**) are representative of at least 3 independent experiments. Significance was calculated using a student T-test. NS: Not significant, * P < 0.05, ** P < 0.01 and *** P < 0.0010

To determine whether the loss of CD109 affects IL6Rα expression, we first analyzed IL6Rα protein expression. We used two types of (vulvar and oral) SCC cells with and without CD109 deletion. Analysis of IL6Rα expression in WT A431 and WT SCC9 cells, and their corresponding CD109 KO cells in the absence and presence of exogenous IL-6 treatment, shows that IL6Rα protein levels are dramatically decreased in CD109 KO cells versus WT cells (Fig. 1A, B, Figure S1A, B). These results suggest that CD109 is an essential regulator of IL6Rα levels through increased synthesis or by decreasing degradation.

The decreased expression of IL6Rα in CD109 KO A431 and CD109 KO SCC9 cells was associated with an abrogation in IL-6-mediated phosphorylation of STAT3 (Tyr 705) as compared to WT A431 and WT SCC9 cells, while total STAT3 levels remained unaffected (Fig. 1A, B, Figure S1A, B). To determine the time course of IL-6-induced STAT3 activation in SCC cells, we examined the phosphorylation of STAT3 at different time points after treatment with IL-6 (Fig. 1C). WT A431 cells showed maximal STAT3 (Tyr 705) phosphorylation after 15 min of treatment which was sustained for up to 30 min and then reduced to ∼50% of maximum after 1 h. In contrast, CD109 KO A431 cells responded weakly to IL-6 with minimal STAT3 phosphorylation (Fig. 1C, Figure S1C). These results were confirmed by immunofluorescence microscopy that demonstrated significantly decreased staining of basal and IL-6-induced phosphorylation of STAT3 (Tyr 705) in CD109 KO A431 cells as compared to WT A431 cells which showed robust nuclear staining of phosphoSTAT3 (Fig. 1D). These results demonstrate that CD109-mediated increase or stabilization of IL6Rα levels results in enhanced STAT3 signaling.

### CD109 and IL6Rα expression in SCC Cells is interdependently regulated

We next examined whether the expression of CD109 and IL6Rα is interdependent by determining the reciprocal regulation of CD109 expression by IL6Rα (Fig. 1E, F, Figure S1D–F). Interestingly, silencing IL6Rα expression in WT A431 cells had significantly reduced the expression of CD109 and phosphoSTAT3 levels in the presence or absence of IL-6 compared to control cells in which IL6Rα is not silenced. As expected, CD109 KO cells acted as controls and no difference was observed between IL6Rα siRNA-silenced and control siRNA-treated CD109 KO cells (Fig. 1E, Figure S1D, E). We next examined the effect of blocking IL6Rα using the FDA-approved humanized monoclonal anti-IL6R alpha antibody, tocilizumab, on the expression of CD109, in two SCC cell lines, A431 and SCC9. Following exposure to tocilizumab at a concentration of 10 µg/ml, there was a significant reduction in the expression of CD109, and phosphoSTAT3, as compared to treatment with the isotype IgG control, in both SCC9 and A431 cells (Fig. 1F, Figure S1F). These findings indicate that the levels of IL6Rα and CD109 are interdependently regulated.

### CD109 forms a complex with IL6Rα and colocalizes with CD109 in SCC cells in an IL-6 ligand-dependent manner

**Fig. 2:**
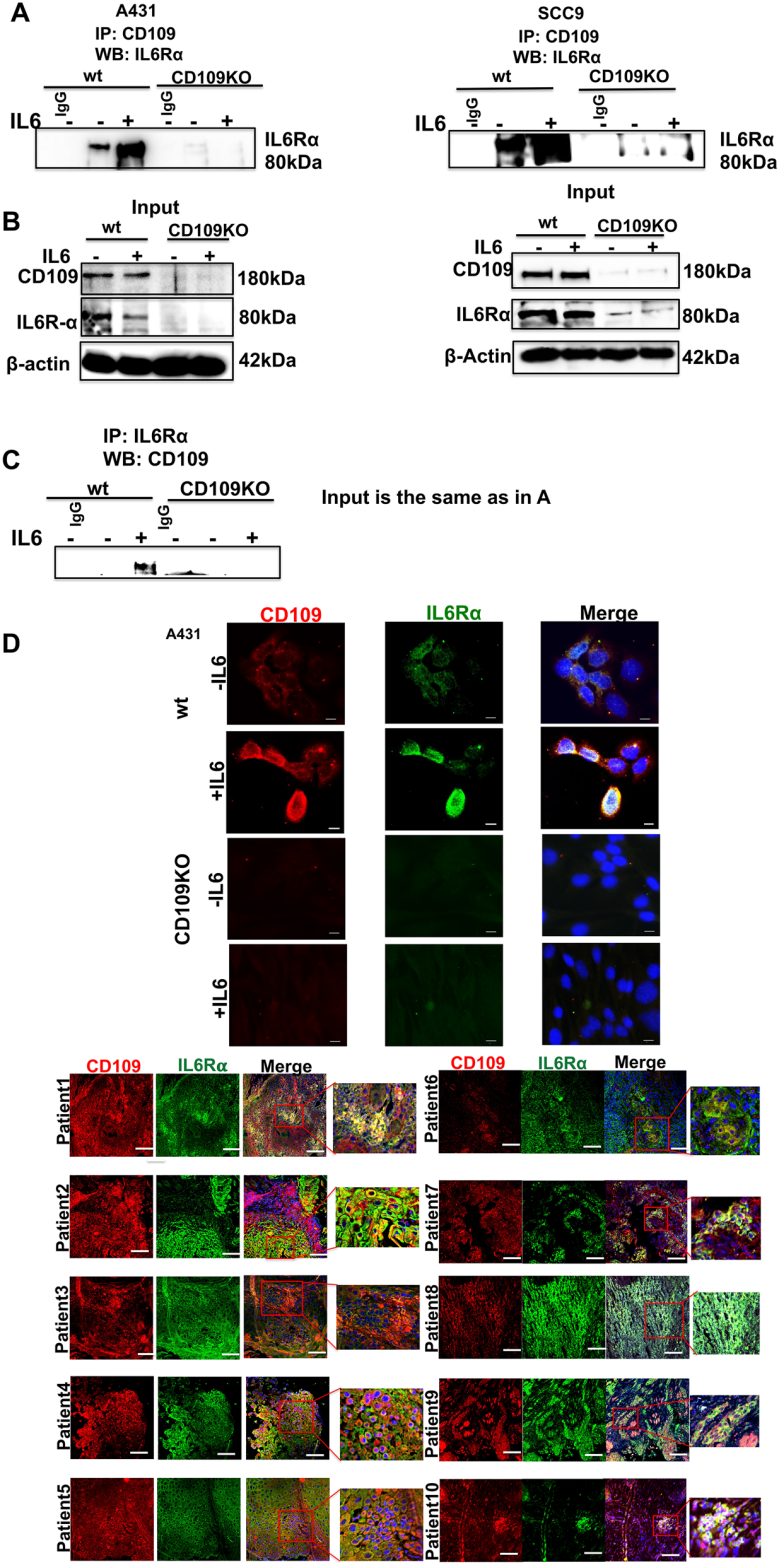
CD109 interacts and co-localizes with IL6Rα in an IL-6-dependent manner. **A** Immunoprecipitation of total cell lysates from A431-WT versus A431-CD109 KO, and SCC9-WT versus SCC9-CD109 KO cells was done using an anti-CD109 antibody or a control IgG followed by Western blot analysis using an anti-IL6Rα antibody. **B **The input data showing the levels of CD109 and IL6Rα in the total cell lysates used are shown below with β-Actin as loading control. **C** Reverse co-immunoprecipitation was performed by immunoprecipitation of total cell lysates from A431-WT versus A431-CD109 KO cells using an anti-IL6Rα antibody or a control IgG followed by Western blot analysis using an anti-CD109 antibody. The input data are same as for panel A. **D** Representative fluorescence microscopic images showing co-expression and colocalization of CD109 and IL6Rα in A431 cells and oral SCC patient derived tissue. CD109 (red), IL6Rα (green) and DAPI (blue) are shown. All data shown **A**–**D** are representative of three independent experiments. Scale bar (**D**): 25 μm

To determine whether CD109 associates with IL6Rα, we conducted co-immunoprecipitation (co-IP) analyses using anti-CD109 antibodies on cell extracts from WT and CD109 KO A431 and SCC9 cells. Our findings revealed that IL6Rα co-immunoprecipitated with CD109 in both WT A431 and WT SCC9 cells, indicating a strong interaction that is dependent on IL-6 (Fig. 2A, B). Conversely, no IL6Rα protein was co-immunoprecipitated in CD109 KO cells (Fig. 2A, B). Reciprocal co-IP experiments showed CD109 co-immunoprecipitation with IL6Rα in WT A431 cells but not in CD109 KO A431 cells (Fig. 2C). Together, these experiments demonstrated a robust CD109-IL6Rα interaction in WT cells, which was absent in CD109 KO cells.

Additionally, we studied the association pattern of CD109 with the IL6Rα-GP130 complex by knocking down GP130 or IL6Rα and observing CD109 interaction changes using co-immunoprecipitation. Knockdown of GP130 disrupted CD109-IL6Rα interaction and knockdown of IL6Rα disrupted CD109-GP130 interaction (Figure S11), suggesting that CD109 likely associates with the IL6Rα-GP130 complex.

Subsequently, we used immunofluorescence microscopy to study the subcellular localization and colocalization of CD109 and IL6Rα in SCC cells and oral SCC patient tumors. CD109 is coexpressed and colocalized with IL6Rα at the cell surface in WT A431 cells, but not on the CD109 KO cells and in oral SCC patient tumors (Fig. 2D). IL-6 stimulation significantly enhanced CD109 and IL6Rα fluorescence intensity in WT A431 cells with no effect in CD109 KO cells, indicating IL-6-dependent enhancement of CD109-IL6Rα association.

### CD109 stabilizes IL6Rα levels on the cell surface and blocks IL6Rα degradation in SCC cells

**Fig. 3:**
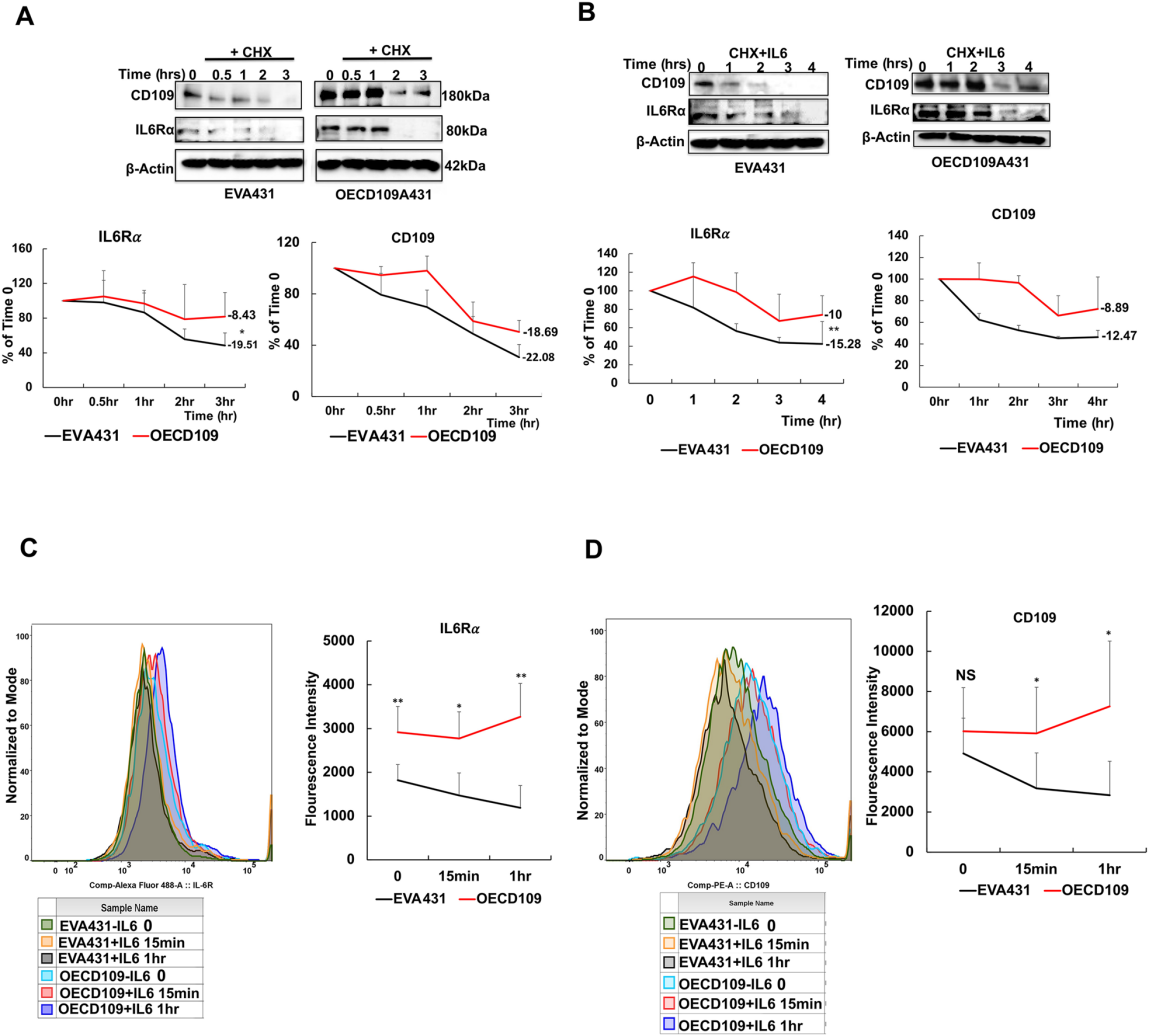
CD109 inhibits IL6Rα degradation and stabilizes IL6Rα levels in SCC cells. **A**, **B** A431 cells overexpressing CD109 (OECD109-A431) or transfected with empty vector (EVA431) were incubated with cycloheximide (CHX, protein synthesis inhibitor) or DMSO (control) for indicated time periods in the absence (**A**) or presence (**B**) of IL-6 (20 ng/ml, added during the last 30 min of incubation) and then expression levels of CD109, IL6Rα and β-Actin was assessed. Densitometric analysis of the data in A and B are shown in the bottom panels. Differences in slopes of the curves representing the degradation data were determined using t-statistics. **C**, **D** Flow cytometric analysis of EVA431 and OECD109-A431 cells treated with IL-6 for 15 min or 1 h and then stained with fluorescein-labeled anti-CD109 (PE-CD109) or anti-IL6Rα (Alexa488-IL6Rα) antibodies. Data shown are the mean ± SD from three independent experiments. Significance between EVA431 and OECD109-A431 cells at different time points was calculated using a Student’s T-test: NS: Non-significant, * P < 0.05. ** P < 0.01 and *** P < 0.001

We next examined the mechanism by which CD109 may regulate IL6Rα expression by conducting a cycloheximide chase assay to monitor IL6Rα degradation kinetics in SCC cells. A431 cells with stable CD109 overexpression (CD109 OE-A431) or empty vector (EV-A431) were treated with the protein synthesis inhibitor cycloheximide, with or without IL-6, for varying durations (0, 1, 2, and 3 h). Western blot analysis revealed that CD109 overexpression blocks IL6Rα degradation, maintaining its cellular levels both under basal conditions and in the presence of IL-6 (Fig. 3A, B). The cytotoxicity of cycloheximide was assessed using MTT assay (Figure S6C).

Furthermore, we determined whether CD109 regulates IL6Rα levels on the cell surface in SCC cells, using flow cytometry (Fig. 3C, D). Cells overexpressing CD109 (OE-A431) exhibited significantly higher levels of cell surface IL6Rα compared to control EV-A431 cells, both under basal conditions and with IL-6 treatment. The cell surface expression of CD109 paralleled that of IL6Rα. These findings suggest that CD109 plays a crucial role in regulating cell surface IL6Rα levels.

### CD109 enhances the IL-6-induced stem cell marker expression and tumorigenicity in SCC cells

**Fig. 4:**
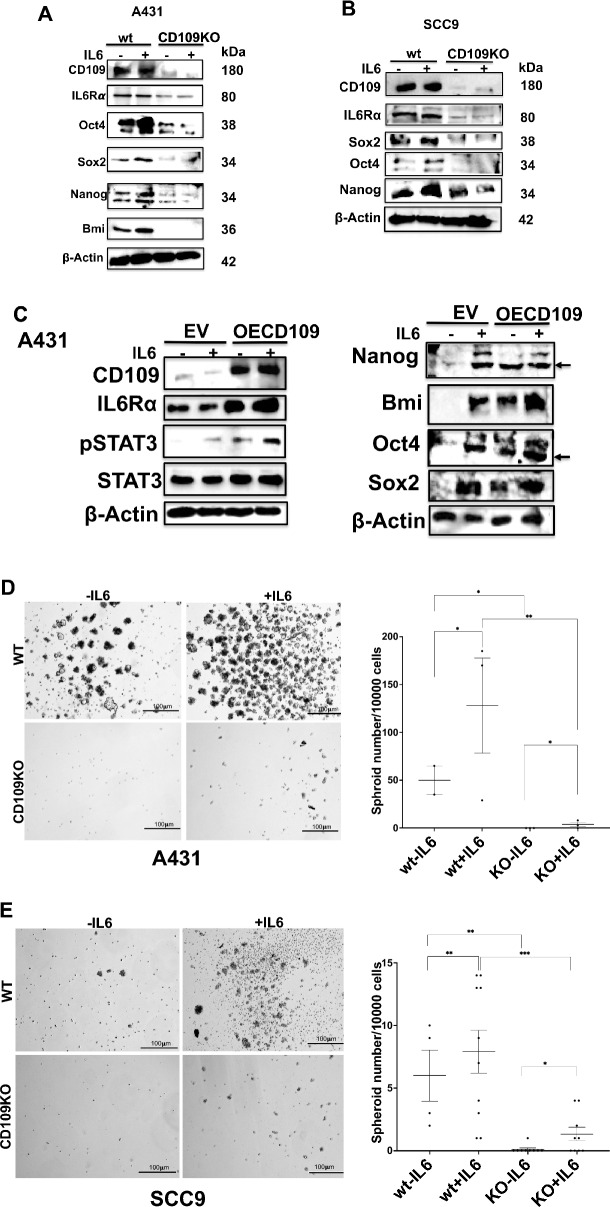
CD109 promotes IL-6-induced expression of stem cell markers and tumorigenicity in SCC cells. **A** A431-WT versus A431-CD109 KO and **B** SCC9-WT versus SCC9-CD109 KO and **C** EV-A431 versus CD109OE-A431 cells were treated with 20 ng/ml of IL-6 for 48 h and Western blot analysis for the indicated stem cell markers was performed. Densitometric analysis of the data depicted in A, B and C are shown in supplementary data. **D**, **E** Tumor spheroids were grown using A431-WT versus A431-CD109 KO (**D**) and SCC9-WT versus SCC9-CD109 KO (**E**) cells to assess in vitro tumorigenicity as described in Methods, and the spheroids formed were counted under the microscope. The percentage of cells capable of forming spheroids was calculated as (number of spheroids formed/number of cells plated) × 100. Quantitation of the results shown in **D** and **E** are shown at the bottom panels. Scale bar: 100 μm. Significance between either wt-IL6 and KO-IL6 or wt + IL6 and KO + IL6 or EVA431-IL6 and OECD109A431-IL6 or EVA431 + IL6 and OECD109A431 + IL6 was calculated using a Student’s T-test: NS: Non-Significant, * P < 0.05, ** P < 0.01 and *** P < 0.001

Our findings mentioned above indicate that CD109 positively influences IL6Rα levels and promotes STAT3 signaling. Given the known role of the IL-6/STAT3 pathway in enhancing cancer cell stemness, it was of interest to explore whether CD109-mediated IL6Rα/STAT3 pathway activation leads to increased stemness. We examined the impact of CD109 on IL-6-mediated stemness in A431 and SCC9 cells, both with and without CD109 deletion, by assessing stem cell marker expression (Sox2, Oct4, Nanog, Bmi1). Deletion of CD109 resulted in reduced basal and IL-6-induced stem cell marker expression in both cell lines (Fig. 4A, B, Figure S2A, B). We also determined whether CD109 regulates IL-6-mediated stem cell marker expression in SCC cells overexpressing CD109. CD109 overexpression increased basal and IL-6-induced stem cell marker expression (Fig. 4C, Figure S2C). These findings highlight CD109's role in enhancing IL-6-induced stemness in SCC cells.

Furthermore, we investigated whether CD109 affects IL-6-induced tumorigenicity in SCC cells using an in vitro tumor spheroid formation assay. Addition of IL-6 to spheroid medium significantly increased spheroid formation in control WT SCC cells but minimally affected CD109 KO cells. CD109 KO cells showed decreased survival and formed smaller, less viable spheroids compared to WT cells (Fig. 4D, E), suggesting CD109's importance in promoting IL-6-induced tumorigenicity.

### CD109 enhances the expression of IL-6-induced NRF2 and its target genes SOD1 and HO1 in SCC cells

NRF2, a key transcription factor, plays a vital role in cellular defence against inflammation and oxidative stress, contributing to cancer cell survival and therapy resistance. Given recent findings linking IL-6-mediated STAT3 activation to NRF2 expression and signaling in cancer progression, particularly in pancreatic and oral SCC, we investigated whether CD109 regulates IL-6-mediated NRF2 expression and its downstream targets SOD1 and HO1 which are crucial anti-oxidative enzymes involved in cell survival and therapy resistance.

**Fig. 5:**
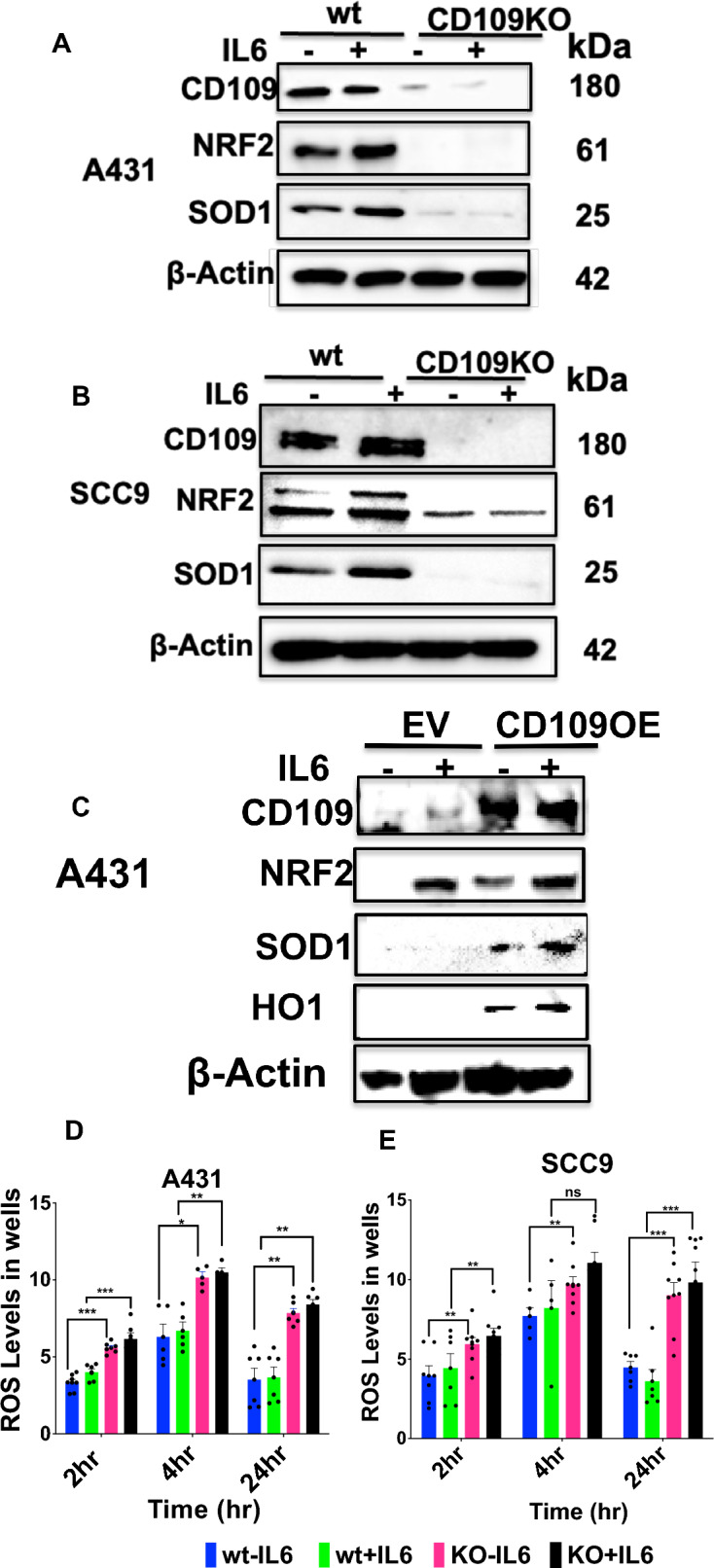
CD109 promotes basal and IL-6-induced expression of antioxidant protein NRF2 and its target genes. **A** A431-WT versus A431-CD109 KO cells and **B** SCC9-WT versus SCC9-CD109 KO cells and **C** EV-A431 and OE CD109A431 cells were treated without or with 20 ng/ml of IL-6 for 24 h. Expression levels of NRF2, SOD1 and HO-1 were analyzed by Western blot. Densitometric analysis of the data depicted in A, B and C are shown in supplementary data. **D**, **E** Measurement of ROS Levels in A431-WT versus A431-CD109 KO and SCC9-WT versus SCC9-CD109 KO cells. Cells were treated without or with 50 ng/ml of IL-6 for 2, 4 and 24 h. ROS levels were measured as described in materials and methods. All the results are expressed as mean ± S.D. of three independent experiments. Significance between either WT-IL6 and KO-IL6 or WT + IL6 and KO + IL6 or EVA431-IL6 and OECD109A431-IL6 or EVA431 + IL6 and OECD109A431 + IL6 was calculated using a student T test: NS: Not-significant * P < 0.05, ** P < 0.01 and *** P < 0.001

We determined whether CD109 regulates IL-6-mediated NRF2 and SOD1 expression using the two SCC cell lines, A431 and SCC9 without and with CD109 deletion, as above. Deletion of CD109 resulted in significantly reduced basal and IL6-induced NRF2 and SOD1 expression in both cell types (Fig. 5A, B, Fig. S3A, B). Conversely, CD109 overexpression increased basal and IL-6-induced NRF2 and SOD1 expression (Fig. 5C, Fig S3C). IL-6 treatment enhanced NRF2, SOD1, and HO1 expression in control cells but not in CD109-deleted SCC cells. Furthermore, IL-6 dose-dependently activated NRF2 in both A431 and SCC9 cells (Figure S7). We further analyzed whether CD109 regulates reactive oxygen species (ROS) levels using A431 WT and SCC9 WT and their corresponding CD109 KO cells. ROS levels were significantly elevated in CD109 knockout (CD109 KO) cells and IL-6 treatment further enhanced ROS levels in both wild-type (WT) and CD109 KO cells (Fig. 5D, E). Together these results illustrate that CD109 significantly enhances IL-6-induced NRF2 and SOD1 expression, leading to suppression of ROS levels in SCC cells. These results support the notion that CD109 promotes therapy resistance in SCC cells.

### STAT3 plays an essential role in the regulation of NRF2 expression

**Fig. 6:**
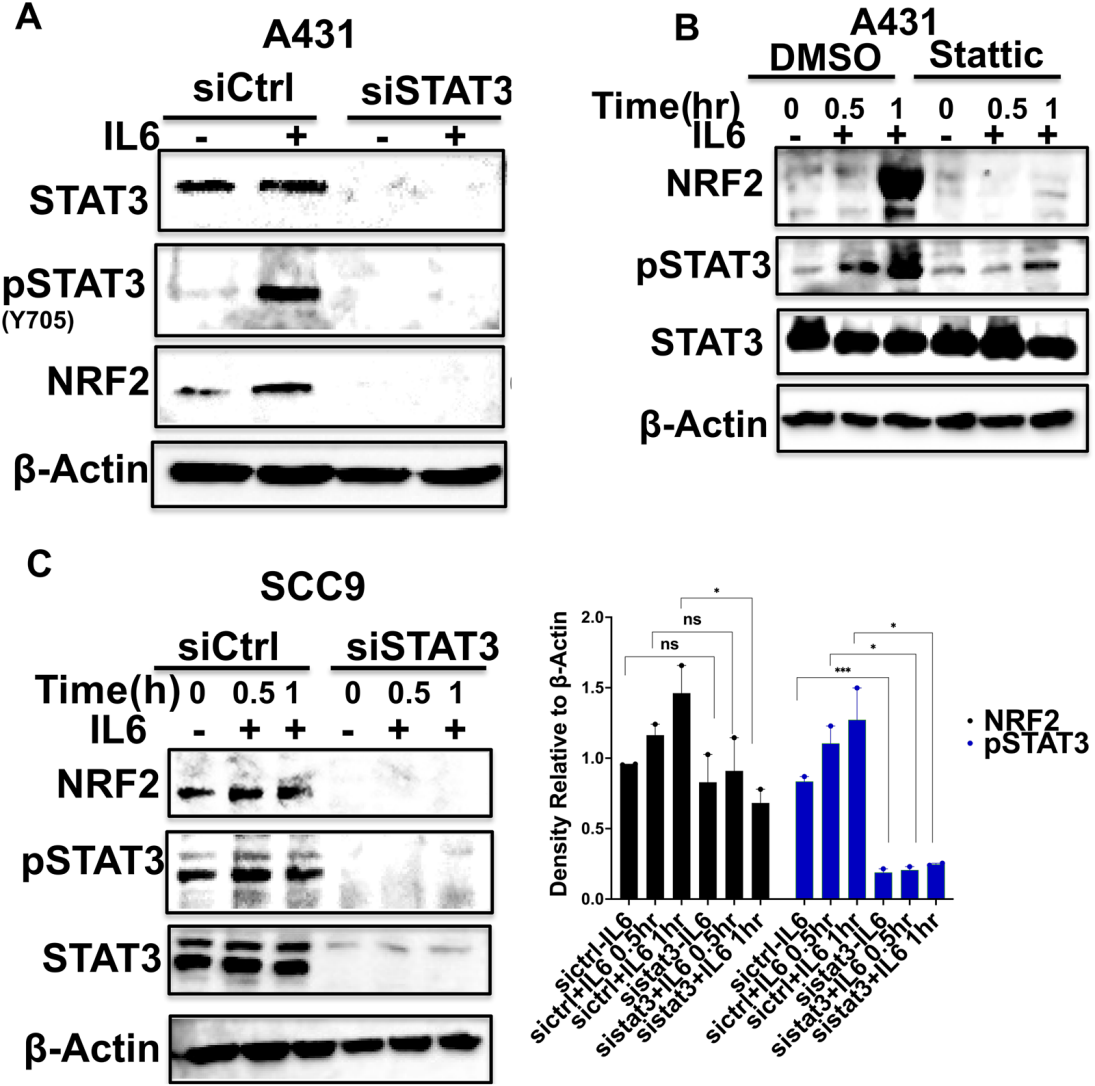
STAT3 is critical for NRF2 protein expression. A431 (**A**) and SCC9 (**C**) cells were transfected with STAT3-specific siRNA for 72 h and were treated with IL-6 (20 ng/ml) for 30 min or 1 h. The levels of NRF2, phospho-STAT3, total STAT3 were analyzed by Western blot. **B** A431 cells were treated with Stattic for 1 h followed by treatment with IL-6 (20 ng/mL) for 30 min or 1 h and the levels of NRF2, phospho-STAT3, total STAT3 were analyzed by Western blot. The quantitation of this data is shown on the left bottom panel. Significance between siCtrl-IL6 and siSTAT3-IL6 or siCtrl + IL6 and siSTAT3 + IL6 0.5 h or siCtrl + IL6 and siCtrl + IL6 1 h was calculated using a student T test: NS: non-significant, * P < 0.05, ** P < 0.01 and *** P < 0.001

To determine whether IL-6-induced STAT3 is directly involved in NRF2 expression in SCC cells, we examined the effects of siRNA-mediated knockdown of STAT3 expression and of inhibiting STAT3 activation and nuclear translocation by a specific STAT3 inhibitor Stattic. Notably, STAT3 knockdown led to a significant decrease in NRF2 protein levels in SCC9 and A431 cells (Fig. 6A, B, Figure S4). Additionally, treatment with Stattic resulted in a pronounced reduction in NRF2 expression in A431 cells (Fig. 6C), affirming the role of the STAT3 pathway in sustaining NRF2 levels in SCC cells. Cell toxicity assessment using MTT assay demonstrated no significant cell death across all Stattic concentrations tested (Figure S6B).

### STAT3 complexes with NRF2 in SCC cells and the IL-6-induced NRF2 expression is crucial for CD109-dependent tumorigenicity

**Fig. 7:**
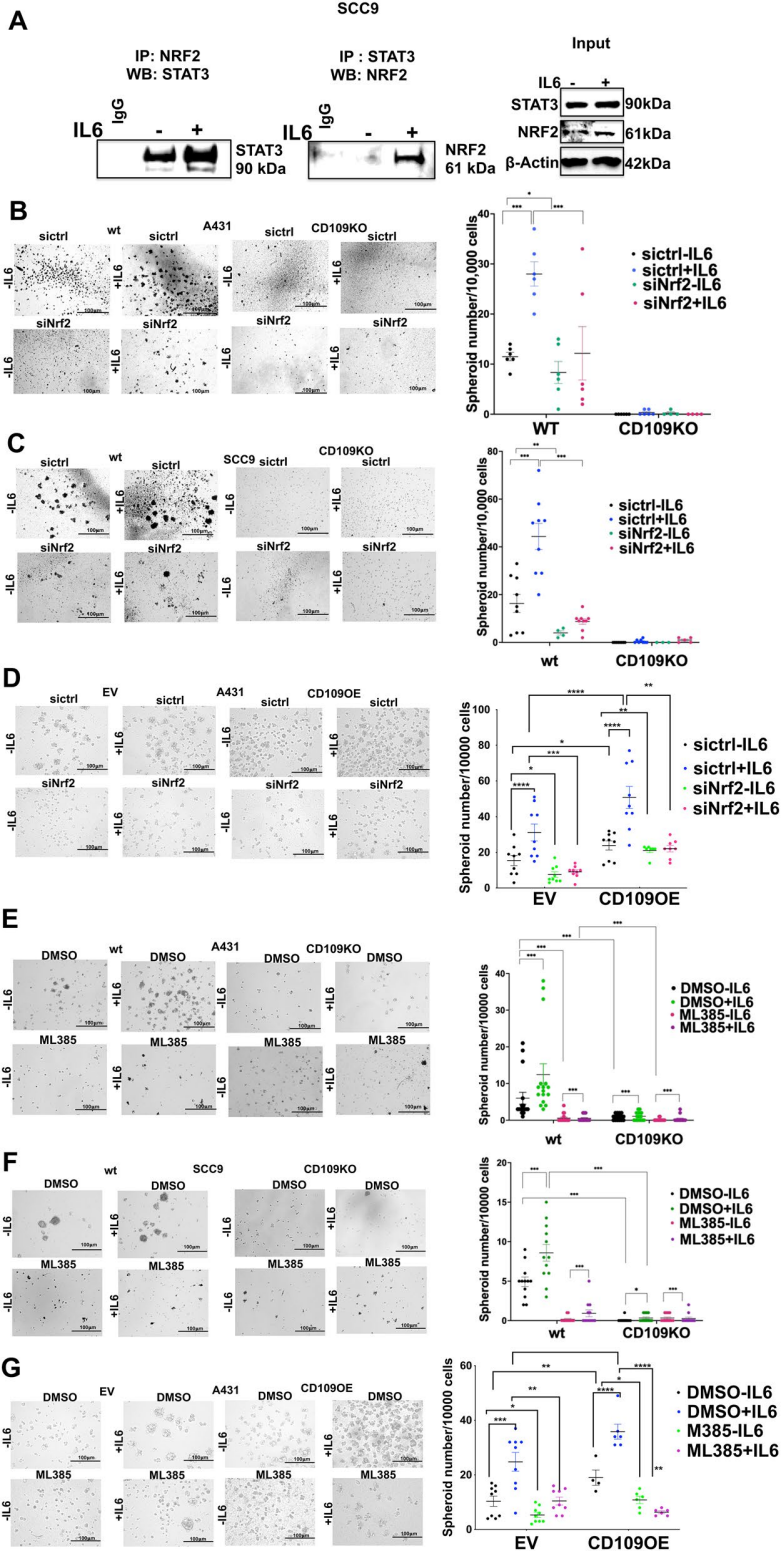
NRF2 associates with STAT3 in an IL-6-dependent manner, mediating the IL-6-CD109 driven SCC stemness. **A** SCC9 cells were incubated with IL-6 (20 ng/ml) overnight and lysates were immunoprecipitated with an anti-NRF2 antibody or control IgG followed by Western blot with an anti-STAT3 antibody (left panel). Reverse co-immunoprecipitation was performed by immunoprecipitating the lysates with an anti-STAT3 antibody followed by Western blot with anti-NRF2 antibody or control IgG (right panel). The input data showing the levels of STAT3 and NRF2 in the total cell lysates used are shown below with β-Actin as loading control. **B**–**G** NRF2 knockdown was performed in A431-WT versus A431-CD109 KO (**B**), or in SCC9-WT versus SCC9-CD109 KO cells (**C**), or in EVA431 versus CD109OE A431 cells (**D**), by transfecting with NRF2-specific siRNA (or control siRNA). Alternatively, NRF2 function was inhibited using ML385, an inhibitor of NRF2, in A431-WT versus A431-CD109 KO (**E**), or in SCC9-WT versus SCC9-CD109 KO cells (**F**) or in EVA431 versus CD109OE A431 cells (**G**). A spheroid formation assay was then performed as described in Methods. The number of tumor spheroids formed was quantified by analyzing five random fields. Scale bar: 100 μm. All the results are expressed as the mean ± S.D. of three independent experiments. Significance was calculated using a Student’s T-test: * P < 0.05, ** P < 0.01 and *** P < 0.001

Given the recent reports suggesting an interaction between NRF2 and STAT3 in breast cancer cells [21], we aimed to determine if a similar association exists in SCC cells. Coimmunoprecipitation studies reveal an IL-6-dependent formation of a complex between STAT3 and NRF2 in SCC9 cells (Fig. 7A), indicating that STAT3 regulates both NRF2 expression and function in SCC.

To confirm that IL-6-induced NRF2 plays an essential role in CD109-controlled stemness of SCC cells, we performed a spheroid formation assay using SCC cells (A431 and SCC9) without and with CD109 deletion and in EV and CD109OE A431 cells. We suppressed NRF2 expression via siRNA-mediated knockdown or inhibited NRF2 activity using a specific inhibitor ML385 and evaluated spheroid formation with or without IL-6 treatment. Results depicted in Fig. 7B, C demonstrate a significant reduction in the number of spheroids formed upon NRF2 knockdown in both A431 and SCC9 cells. In contrast, the number of spheroids formed was significantly higher in CD109OE as compared to EV cells (Fig. 7D). Similarly, treatment with ML385 led to a marked decrease in spheroid formation (Fig. 7E–G), indicating that basal and IL-6-induced NRF2 are crucial for CD109-dependent pro-tumorigenic activity. Confirmation of ML385 cytotoxicity was performed using an MTT assay (Figure S6A).

### CD109 protein and mRNA levels correlate with those of IL6Rα, phopshoSTAT3, NRF2 and NRF2 target gene expression in oral SCC and HNSCC patient tumors

**Fig. 8:**
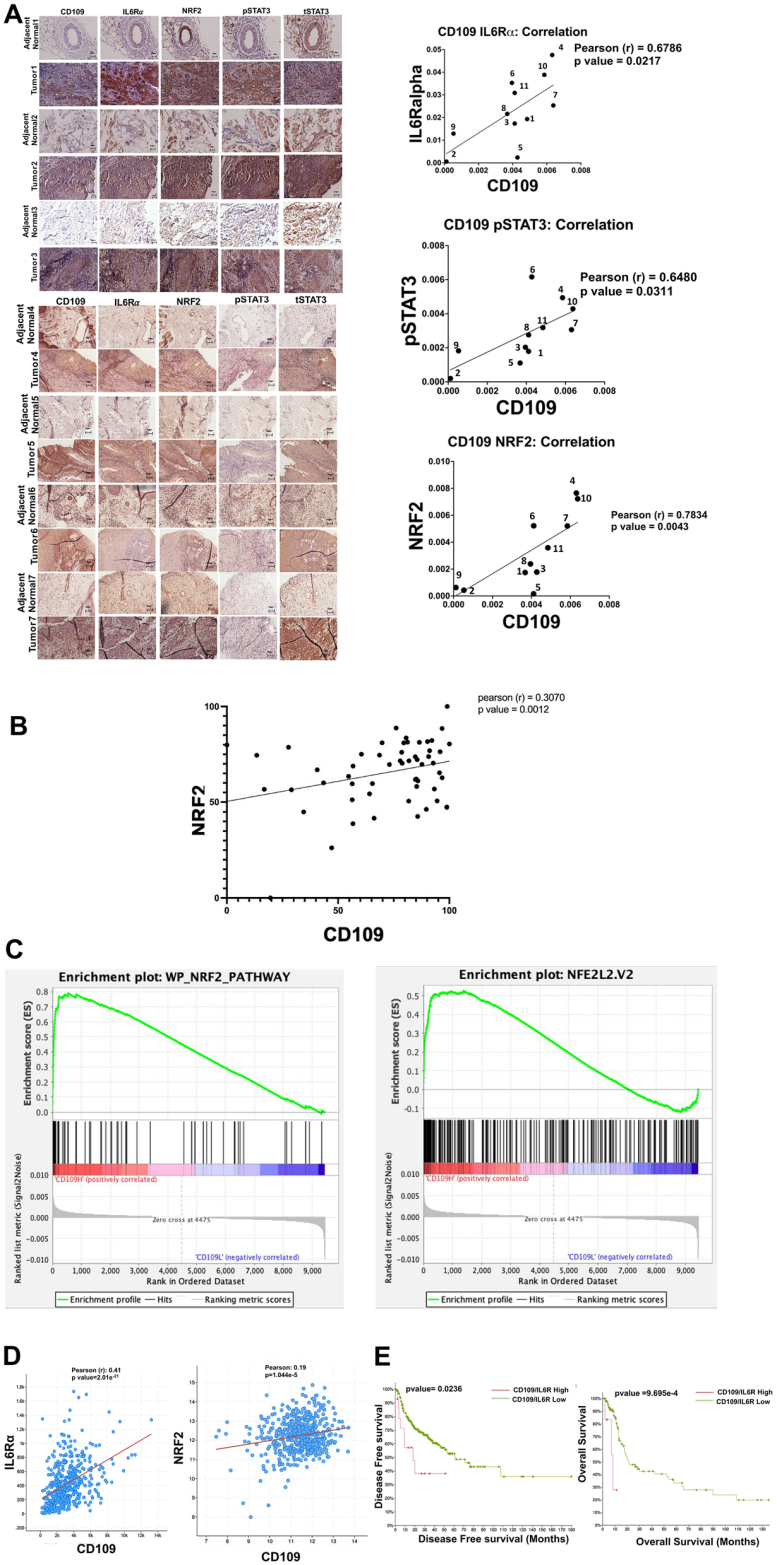
**CD109 expression correlates positively with the levels of IL6Rα, NRF2, phospho-STAT3 and negatively with survival. (A)** Immunohistochemical staining for CD109, IL6Rα, NRF2, phospho-STAT3 and total STAT3 was performed on paraffin-embedded oral SCC tumor tissues (n=15) and normal adjacent tissues (n=11) from a total of fifteen patients. Data for seven patients are shown (see supplementary figure 8 for additional data). CD109 protein expression was correlated with IL6Rα, NRF2 and pSTAT3 levels using ImageJ analysis (n=11). Staining intensity was normalized to that of adjacent normal samples. Pearson’s correlation analysis was done to determine the coefficient (r value) and statistical significance was assessed using a t-test. Scale bars: 50 μm. **(B)** Analysis of HNSCC patient data obtained from LinkedOmics data base (http://www.linkedomics.org) shows that protein expression of CD109 correlates with that of NRF2 as determined by Pearson correlation analysis. **(C)** CD109 protein expression correlates with NRF2 pathway activation as analyzed by Gene Set Enrichment Analysis (GSEA) of data from the LinkedOmics data base. **(D)** CD109 mRNA expression also correlates with IL6Rα and NRF2 expression in 523 HNSCC patients from the TCGA Pancancer Atlas database (cBioPortal, https://www.cbioportal.org/) as determined by Pearson’s correlation analysis. **(E)** CD109/IL6Rα expression negatively correlates with disease-free and overall survival in HNSCC patients. The 523 patients were stratified into CD109 high/IL6Rα high and CD109 low/IL6Rα low groups, and survival probability was assessed by a log-rank (Mantel-Cox) test

To determine whether our in vitro findings align with the in vivo situation, we next measured using immunohistochemistry, the protein levels of CD109, IL6Rα, pSTAT3 and NRF2 in SCC tumors and adjacent normal tissue collected from fifteen oral SCC patients at surgery. Our results show that the levels of CD109, IL6Rα, pSTAT3 and NRF2 are markedly upregulated in tumor sections compared to normal tissue and CD109 levels correlate significantly with those of IL6Rα/pSTAT3/NRF2 (Fig. 8A, Figure S8). Clinical Data of the patients who consented to provide samples for this study are summarized in Supplementary Table 1.

Furthermore, we corroborated our findings above with a larger number (n = 109) of patients by proteomic analysis of data on HPV negative HNSCC patients from the LinkedOmics database and dividing them into CD109 high versus CD109 low groups (see Methods). Our findings show that CD109 protein expression significantly correlates with NRF2 protein expression (Fig. 8B). In addition, gene set enrichment analysis against the molecular signatures database (see Methods) using the same proteomic data involving 9666 identified proteins reveal that CD109 protein expression correlates with NRF2 pathway activation (levels of NRF2 target proteins) (Fig. 8C, Figure S9, Supplementary Table 2). (Data on IL6Rα protein expression was unavailable in the linkedOmics data set to examine its correlation with CD109 protein expression).

Further confirmation of our findings using gene expression analysis of 523 HNSCC patients data available at the cBioPortal for Cancer Genomics/TCGA database (see Methods) reveal that CD109 mRNA expression correlates with the expression of IL6Rα and NRF2 mRNA (Fig. 8D). In addition, our qPCR results using SCC cells with CD109 overexpression or CD109 deletion show that CD109 strongly regulates the expression of IL6Rα, NRF2, and NRF2 target genes (HO1, SOD1, and SOD2) (Figure S10 A, B).

We also analyzed the TCGA data used above to determine the relationship between CD109/IL6Rα expression levels and overall and disease-specific patient survival probabilities in HNSCC patients. Our results show that high expression of CD109 and IL6Rα (CD109^high^/IL6Rα^high^ versus CD109^low^/IL6Rα^low^) correlated with worse overall and disease-free survival, suggesting that CD109 represents an important prognostic marker in HNSCC patients (Fig. 8E).

## Discussion

Squamous cell carcinoma (SCC) is a prevalent malignancy with poor prognosis, causing over 330,000 deaths annually. Understanding the molecular mechanisms driving SCC development and progression is crucial for effective treatment strategies. We have previously identified CD109 as a TGF-β co-receptor and potent antagonist of TGF-β signaling [11] and have reported that it inhibits TGF-β-induced fibrotic parameters [2, 27, 53, 54, 56] and epithelial to mesenchymal transition [61], suggesting that CD109 may have anti-tumorigenic properties. Paradoxically, however, CD109 is upregulated in SCC and we and others found that it strongly promotes SCC tumor progression via EGFR pathway activation [32, 62]. It remains unclear whether CD109 controls other oncogenic signaling pathways crucial for SCC progression. Here we show that CD109 plays a pivotal role as an essential regulator of IL6Rα expression and IL-6-driven oncogenic processes, enhancing cancer cell stemness and antioxidant capabilities in SCC cells.

We show that loss of CD109 leads to a significant decrease in cell surface and total IL6Rα protein levels, leading to loss of stemness in SCC cells. Furthermore, CD109 enhances IL-6-induced STAT3 signaling via IL6Rα, promoting STAT3-NRF2 interaction and expression of antioxidant proteins leading to the suppression of ROS levels, suggesting a crucial role for CD109 in controlling IL6Rα-mediated cellular detoxification and thus resistance to therapy. Consistent with these in vitro findings, in vivo immunohistochemical analysis of oral SCC tumor samples, and correlation studies of publicly available HNSCC patient data for mRNA (cBioportal for Cancer Genomics) and proteomic (LinkedOmics) expression reveal that CD109 expression positively correlates with the activation of the IL6Rα/STAT3/NRF2 pathway. The proteome data involving 109 HNSCC patients with 9666 identified proteins in the Linkedomics data set (http://www.linkedomics.org) revealed a significant positive correlation between CD109 and NRF2 expression and pathway activation. IL6Rα protein expression data was not available in the Linkedomics database. However, our immunohistochemical (IHC) analysis of fifteen oral SCC patient tumors revealed a significant positive correlation between CD109 and IL6Rα expression. Nevertheless, establishing a clear association between CD109/IL6Rα positivity and patient prognosis would require a larger cohort.

This finding was corroborated with genomics data involving 523 HNSCC patients in the cBio Portal for Cancer Genomics data set, showing a clinically relevant positive correlation of CD109 expression with the expression of IL6Rα, NRF2 and NRF2 target genes. These findings were further validated by qPCR analysis (Figure S6) and microarray data (not shown) demonstrating a significant decrease in NRF2 expression in SCC cells with and without CD109 deletion. Taken together, our findings identify a novel CD109/IL6Rα/STAT3/NRF2 axis as central to SCC progression, suggesting an essential role for CD109 in IL-6-mediated oncogenic process in SCC.

In the current study we show that the mechanism by which CD109 controls IL6Rα cell surface levels and signaling involves ligand-dependent interaction and colocalization with IL6Rα protein, thereby delaying IL6Rα degradation, leading to IL6Rα stabilization on the cell surface and enhanced IL6Rα-mediated signaling and responses. The inference that CD109-IL6Rα interaction leads to stabilization of IL6Rα on the cell surface is supported by results from protein degradation assay in the presence of cycloheximide and by FACS analysis which demonstrate that CD109 regulates the cell surface levels of IL6Rα as early as 15 min after IL-6 treatment. Interestingly, our data suggest that CD109 and IL6Rα exist on the cell surface as a pre-formed receptor complex and that this interaction is strengthened by IL-6 ligand. Whether IL-6 binds solely IL6Rα or whether it binds simultaneously to both CD109 and IL6Rα within a pre-existing complex to strengthen the IL6Rα-CD109 interaction remains to be determined. These findings indicate that the marked reduction of IL6Rα protein in CD109 knockout cells as compared to wild-type cells, to a large extent, is due to the loss of CD109-IL6Rα interaction, resulting in targeting of IL6Rα for degradation.

It was recently reported that CD109 interacts with GP130, a component of the IL-6 receptor complex, in glioblastoma cells [10]. As gp130 participates in the signaling of many members of the IL-6 family of cytokines, including IL-6, IL-11, IL-17, leukemia inhibitory factor (LIF), ciliary neurotrophic factor (CNTF), oncostatin M (OSM), among others[30], it is important to establish whether CD109 regulates GP130-mediated IL6Rα signaling. Our study reveals that CD109 interacts with both gp130 and IL6Rα, with IL6Rα knockdown impairing CD109-gp130 interaction, highlighting CD109's role in stabilizing the entire IL-6 receptor complex. These findings together establish CD109’s pivotal role in regulating IL6Rα stability in SCC cells.

STAT3, a transcription factor activated in various human malignancies, including SCC, responds to multiple upstream signals such as IL-6 receptor, EGFR, and other kinases [20]. Although a previous study has shown that CD109 induces EGFR-mediated STAT3 phosphorylation in cervical SCC cells [32], our previous results indicate that CD109 facilitates EGFR/AKT signaling in A431 (vulvar) SCC cells [62]. Our findings are in agreement with another report showing that elevated CD109 levels activate EGFR-AKT-mTOR signaling in lung adenocarcinoma cells [25]. These results together with our current finding that CD109 directly interacts with IL6Rα and strongly promotes IL-6-induced STAT3 signaling, suggest that the regulation of STAT3 signaling by CD109 is mediated predominantly via IL6Rα rather than EGFR in SCC cells, and in addition suggest that CD109 controls the balance of signaling via IL-6/STAT3 versus EGFR/STAT3 in SCC cells.

Our finding that CD109-mediated increases in IL6Rα levels and STAT3 signaling leads to the stimulation of downstream IL-6 responses including stem cell marker expression and tumorigenicity, as well as NRF2 expression and activation of antioxidant enzymes, together with suppression of ROS levels, underscores a crucial role of CD109 in regulating IL-6-mediated cancer cell stemness, antioxidant status, and thus survival of SCC cells. Our finding that the inhibition of NRF2 expression (by siRNA knockdown or the NRF2-specific inhibitor ML385) significantly reduces CD109-mediated IL-6-dependent stemness in SCC cells, support a unique model where the CD109/IL6Rα/STAT3/NRF2 axis plays a critical role in promoting cancer cell stemness.

Interestingly, our observation that STAT3 is associated with NRF2 and forms a complex in an IL-6-dependent manner raises the possibility that this interaction plays a key role in mediating the IL-6-induced expression of the anti-oxidant enzymes HO1 and SOD1 and suppression of ROS levels contributing to therapy resistance in SCC cells. Given that blocking STAT3 expression or activity downregulates NRF2 expression indicates that NRF2 acts downstream of STAT3 activation, although reciprocal regulation cannot be ruled out. While the literature on the interplay between STAT3 and NRF2 is limited, our results are consistent with a previous report showing a significant correlation between STAT3 and NRF2 in breast carcinoma tissues [21]. Our finding underscoring CD109's essential role in regulating the IL-6-induced STAT3/NRF2 pathway supports the emerging evidence that increased IL-6 protein levels confer radio-resistance to SCC cells by preventing oxidative damage via the NRF2-antioxidant pathway [29].

The association of CD109 with IL-6Rα, or its regulation of IL-6Rα/STAT3/ and NRF2 has not been reported and the clinical relevance of such regulation has not been shown previously. In the present study, the clinical data from immunohistochemistry analysis of oral SCC tumors from fifteen different patients reveal a significant positive correlation between CD109 levels and levels of IL6Rα, phosphoSTAT3, and NRF2, all of which are markedly elevated in the oral SCC tumor tissue as compared to adjacent control tissue. Determining whether the expression levels of these proteins correlate with pathological grade and tumor stage is currently in progress. Further clinical validation of the regulation of the IL6Rα and NRF2 pathway by CD109 was obtained using proteomic and genomic analyses of HPV-negative HNSCC patient data from the Linkedomics database and the TCGA dataset at the cBio Cancer Genomics Portal, respectively [3, 12]. Analysis of the proteome data involving 109 HNSCC patients revealed a significant positive correlation between CD109 and NRF2 expression and NRF2 functional pathway, while querying the genomic data comprising 523 HNSCC patients showed a clinically relevant positive correlation between CD109 expression and the expression of IL6Rα, NRF2 and a large number of NRF2 target genes. This is consistent with our qPCR data showing that CD109 deletion leads to a significant decrease in the mRNA expression of IL6Rα, NRF2, SOD2 and HO1 in oral and/or vulvar SCC cells. Together, these findings emphasize the clinical significance of the CD109/IL6Rα/STAT3/NRF2/antioxidant pathway as an important molecular mechanism mediating SCC progression.

Stratifying HNSCC patient data from TCGA into ‘CD109 high/IL6Rα high’ versus ‘CD109 low/IL6Rα low’ reveals worse disease-specific and overall survival (OS) probabilities in ‘CD109 high/IL6Rα high’ patients, suggesting the potential of CD109/IL6Rα co-expression as a prognostic signature for SCC progression. Although high expression of CD109 has previously been reported to be linked to unfavourable disease-free or overall survival in oropharyngeal SCC [51] and across various cancers, including epithelioid sarcoma, breast cancer, and lung adenocarcinoma [24], the significance of CD109 and IL6Rα co-expression in SCC progression hasn't been reported previously.

## Conclusion

In summary, we have identified a novel CD109/IL6Rα/STAT3/NRF2 axis that plays a crucial role in regulating stemness and antioxidant responses in SCC cells. We show for the first time that CD109 interacts with and promotes the stability of IL6Rα on the cell surface, and that this interaction is required for IL-6/STAT3/NRF2 signaling in SCC cells. Our results suggest an essential role for CD109 in IL-6-dependent SCC progression and IL-6/NRF2-mediated resistance to therapy and provide a basis for a distinct avenue for therapeutic intervention in SCC. Also, our data indicate a potential utility for exploring CD109/IL6Rα co-expression as a diagnostic/prognostic tool for SCC progression. Using analysis of oral SCC patient tumors, together with the proteomic and genomic analysis of the HNSCC patient data we reveal an important association of CD109 expression with the expression of IL6Rα, phosphoSTAT3, NRF2 and NRF2 target genes, demonstrating the clinical relevance of the CD109/IL6Rα/STAT3/NRF2/antioxidant pathway as an important molecular mechanism mediating SCC progression.

## Supplementary Information

Supplementary Material 1.

Supplementary Material 2.

Supplementary Material 3.

## Data Availability

No datasets were generated or analysed during the current study.

## Abbreviations

AKR1B10: Aldo–keto Reductase 1B10
AKR1C1: Aldo–keto Reductase 1C1
AKR1C2: Aldo–keto Reductase 1C2
AKR1C3: Aldo–keto Reductase 1C3
Bmi1: B lymphoma Mo-MLV insertion region 1 homolog
Co-IP: Co-immunoprecipitation
CD109: Cluster of Differentiation 109
EGFR: Epidermal growth factor receptor
EMT: Epithelial-Mesenchymal transition
EV: Empty vector
GCLC: Glutamate-cysteine ligase
GCLM: Glutamate-Cysteine Ligase Modifier Subunit
GP130: Glycoprotein 130
gRNA: Guide interfering ribonucleic acid
GSEA: Gene set enrichment analysis
HNSCC: Head and neck squamous cell carcinoma
HO-1: Heme Oxygenase 1
IF: Immunofluorescence
IHC: Immunohistochemistry
IL-6: Interleukin 6
IL6Rα: Interleukin6 receptor alpha
KEAP1: Kelch-like ECH-associated protein 1
KO: Knockout
MTT: 3-(4,5-Dimethylthiazol-2-yl)-2,5-diphenyltetrazolium bromide
NRF2: Nuclear factor erythroid 2-related factor 2
NTRK2: Neurotrophic tyrosine receptor kinase
Oct4: Octamer-binding transcription factor 4
OE: Overexpressing
qPCR: Quantitative polymerase chain reaction
ROS: Reactive Oxygen species
SCC: Squamous cell carcinoma
SD: Standard deviation
SLC7A11: Solute Carrier Family 7 Member 11
siRNA: Small, interfering ribonucleic acid
Sox2: Sex determining region Y-box 2
SOD1, SOD2: Superoxide dismutase 1 and 2
SRXN1: Sulfiredoxin 1
STAT3: Signal Transducer and Activator of Transcription 3
TCGA: The Cancer Genome Atlas
TGF-β: Transforming growth factor beta
TXNRD1: Thioredoxin Reductase 1
Tyr 705: Tyrosine 705
UCHL1: Ubiquitin C-terminal hydrolase 1
WT: Wild type
