# Correction: IL‑6‑mediated tumorigenicity and antioxidant state in squamous cell carcinoma cells are driven by CD109 via stabilization of IL‑6 receptor‑alpha and activation of STAT3/NRF2 pathway

**DOI:** 10.1186/s40164-025-00671-2

**Published:** 2025-05-31

**Authors:** Amani Hassan, Tenzin Kungyal, Shufeng Zhou, Meryem Blati, Kenneth Finnson, Nick Bertos, Nahid Golabi, Nader Sadeghi, Sampath Loganathan, Anie Philip

**Affiliations:** 1 Divisions of Plastic Surgery, and Surgical and Interventional Sciences, Department of Surgery, McGill University, Montreal, QC Canada; 2 Divisions of Dermatology and Experimental Medicine, Department of Medicine, McGill University, Montreal, QC Canada; 3 Divisions of Thoracic and Upper GI Surgery, Department of Surgery, McGill University, Montreal, QC Canada; 4 Department of Otolaryngology, Head and Neck Surgery, Division of Experimental Medicine, McGill University Health Center, Montreal, QC Canada; 5 Montreal General Hospital, 1650 Cedar Avenue, Room C10-148.4, Montreal, QC H3G 1A4 Canada

**Correction to: Experimental Hematology & Oncology (2025) 14:64**

10.1186/s40164-025-00630-x.

In the caption for Fig. 8 of this article [1], an error occurred due to the inadvertent omission of information. The original and corrected captions are provided below. Furthermore, the supplementary material initially published was accurate but inaccessible. This file has now been revised and replaced with a corrected, viewable version.

Incorrect Figure Caption

**Figure 8** CD109 expression correlates positively with the levels of IL6Rα, NRF2, phospho-STAT3 and negatively with survival. **A** Immunohistochemical staining for CD109, IL6Rα, NRF2, phospho-STAT3 and total STAT3 was performed on paraffin-embedded oral SCC tumor tissues and normal adjacent tissues from six patients. Data for three patients are shown. CD109 protein expression was correlated with IL6Rα, NRF2 and pSTAT3 levels using ImageJ and staining intensity was normalized to control samples. Statistical significance was assessed using Pearson’s correlation coefficient (see supplementary Fig. 4 for additional data), Scale bars: 50 μm. **B** Analysis of HNSCC patient data shows that protein expression of CD109 correlates with that of NRF2. Pearson correlation analysis revealed a significant correlation between CD109 and NRF2 protein expression, as analyzed by GraphPad Prism. **C** CD109 protein expression correlates with NRF2 pathway activation in Gene Set Enrichment Analysis (GSEA). **D** CD109 mRNA expression also correlates with IL6Rα and NRF2 expression in 523 HNSCC patients from the TCGA Pancancer Atlas database (cBioPortal, https://www.cbioportal.org/.) as analyzed by Pearson’s correlation test. **E** CD109/IL6Rα expression negatively correlates with disease-free and overall survival in HNSCC patients. The 523 patients were stratified into CD109 high/IL6Rα high and CD109 low/IL6Rα low groups, and survival probability was assessed by a log-rank (Mantel-Cox) test.

Correct Figure Caption

**Figure 8**. **CD109 expression correlates positively with the levels of IL6Rα**,** NRF2**,** phospho-STAT3 and negatively with survival. (A)** Immunohistochemical staining for CD109, IL6Rα, NRF2, phospho-STAT3 and total STAT3 was performed on paraffin-embedded oral SCC tumor tissues (*n* = 15) and normal adjacent tissues (*n* = 11) from a total of fifteen patients. Data for seven patients are shown (see supplementary Fig. 8 for additional data). CD109 protein expression was correlated with IL6Rα, NRF2 and pSTAT3 levels using ImageJ analysis (*n* = 11). Staining intensity was normalized to that of adjacent normal samples. Pearson’s correlation analysis was done to determine the coefficient (r value) and statistical significance was assessed using a t-test. Scale bars: 50 μm. **(B)** Analysis of HNSCC patient data obtained from LinkedOmics data base (http://www.linkedomics.org) shows that protein expression of CD109 correlates with that of NRF2 as determined by Pearson correlation analysis. **(C)** CD109 protein expression correlates with NRF2 pathway activation as analyzed by Gene Set Enrichment Analysis (GSEA) of data from the LinkedOmics data base. **(D)** CD109 mRNA expression also correlates with IL6Rα and NRF2 expression in 523 HNSCC patients from the TCGA Pancancer Atlas database (cBioPortal, https://www.cbioportal.org/) as determined by Pearson’s correlation analysis. **(E)** CD109/IL6Rα expression negatively correlates with disease-free and overall survival in HNSCC patients. The 523 patients were stratified into CD109 high/IL6Rα high and CD109 low/IL6Rα low groups, and survival probability was assessed by a log-rank (Mantel-Cox) test.

The original article has been corrected.
