# Supplementary material for: IL-6-mediated tumorigenicity and antioxidant state in squamous cell carcinoma cells are driven by CD109 via stabilization of IL-6 receptor-alpha and activation of STAT3/NRF2 pathway
Source: Exp Hematol Oncol. 2025 May 2;14:64. doi: 10.1186/s40164-025-00630-x (PMC12046912; doi:10.1186/s40164-025-00630-x)
Supplement: Supplementary file 1 — Supplementary Material 1. [file 40164_2025_630_MOESM1_ESM.docx]

**Supplementary Methods:**

**MTT Assay**

Cells (3x10^4/well) were seeded in a 96-well microtiter plate with 200 μL/well and incubated for 24 hours. Subsequently, cycloheximide, ML385, or Stattic was added. After 4 hours of incubation at 37°C, 20 μL/well of MTS reagent was added and incubated under standard conditions. The plate was briefly shaken, and absorbance was measured at OD=490 nm using a plate reader for both treated and untreated cells.

**RNA Isolation, Reverse Transcription, PCR, and Real-Time PCR**

Total RNA was isolated from A431 WT, CD109 KO, EVA431, and CD109OEA431 cells using TRizol according to the manufacturer's instructions (#15596026, Invitrogen). One microgram of RNA was reverse transcribed into first-strand cDNA using NEB Reverse Transcriptase (#E3010S, LunaScript® RT SuperMix Kit). Gene expression was quantified using a 7900HT Fast Real-Time PCR System (Thermo Fisher Scientific, Toronto, ON, Canada) with Luna® Universal qPCR Master Mix (#M3003L, NEB). The fold change was determined using the delta CT method.

**Supplementary Figures:**

**Supplementary Figure 1: Densitometric analysis of the data depicted in Figure1**

**B**

**A**

**C**

**D**

**C**

**F**

**E**

**F**

**Supplementary Figure 2: Densitometric analysis of the data depicted in Figure 4.**

**A**

**B**

**C**

**A**

**Supplementary Figure 3. Densitometric analysis of the data depicted in Figure 5.**

**B**

**A**

**C**

**Supplementary Figure 4: Densitometric analysis of the data depicted in Figure 6.**

**Supplementary Figure 5. Sequencing analysis of wt and CD109 knockout cell lines generated using CRISPR/Cas9.** (A-B) Sequencing chromatograms display representative sequencing chromatograms for wt and CD109 knockout alleles. The wt sequence is shown with the target gene sequence intact, while the knockout sequence demonstrates the specific mutation (Insertion of T in exon 2) introduced at the target site in both A431 and SCC9 cell lines. The position of the alteration is indicated for clarity. CACCGACTATTGGGGTGGAGCTTC and CTTCCAGGACAGAGACAGTG are two sequences of the gRNAs used in A431 and SCC9 cells.

**Supplementary Figure 6.** **ML385, Stattic, and cycloheximide show no cytotoxicity on SCC cells, as assessed by MTT assay.** ML385 (NRF2 inhibitor) or Stattic (STAT3 inhibitor) or Cycloheximide (a protein synthesis inhibitor) was tested across a range of concentrations to evaluate its impact on cell viability over a 24-hour exposure period as measured by absorbance at 570 nm (OD 570). A) Dose-response curve displays the normalized cell viability relative to untreated control cells in across increasing concentrations of ML385 (0, 2, 5, 10 μm), Stattic (0, 10, 20, 40 μm) and cyloheximide (1, 5, 25, 50, 75 μg/ml). MTT assay was performed as described in Materials and Methods. Each data point represents an average of three replicates.

**Supplementary Figure 7. IL-6 dose-dependently increases NRF2 expression in SCC cells.** Effects of varying concentrations of IL6 on the expression levels of NRF2 in A431 and SCC9 cells. Western blot bands represent the expression levels of NRF2 after treatment with increasing concentrations of IL6 (0, 1, 2.5, 5, 7.5, 10, 25, 50, 75, 100, 200, 300 ng/ml) for 24 hrs. β-Actin was used as a loading control to ensure equal protein loading across samples. The graph shows the normalized expression levels of NRF2 relative to the housekeeping protein, plotted against IL-6 concentrations.

**Supplementary Figure 8. Expression of CD109 positively correlates with that of IL6Rα, NRF2 and pSTAT3 in oral SCC**. Representative images showing the IHC staining of CD109, IL6Rα, NRF2, pSTAT3 and STAT3 in oral SCC tumors collected from patients. All proteins are expressed within the same tissue region. Scale bar 50 μm.

**Supplementary Figure 9: Analysis of TCGA-HNSCC data shows that CD109 expression correlates significantly with 11 NRF2-regulated genes**. Expression signature of 11 genes regulated by CD109-NRF2 axis extracted from TCGA-HNSCC. A scatter plot displaying the normalized expression levels of each gene (x-axis) and CD109 (y-axis) across Head and Neck squamous cell carcinoma patients. The Spearman and Pearson correlation coefficient (R) and the p-value obtained from the correlation analysis are indicated in each plot.

**Supplementary Figure 10: CD109 regulates the mRNA expression of IL6Rα, NRF2, HO1, SOD1 and SOD2.** Quantitative PCR (qPCR) analysis results demonstrating the reciprocal regulation of IL6Rα, NRF2, HO1 and SOD1, SOD2 expression in knockout (KO) and overexpression of CD109 in WT A431 and CD109 KOA431 or EVA431 and CD109OEA431 cells A) Bar graph showing the relative expression levels of IL6Rα, NRF2, HO1 and SOD1, SOD2 mRNA in wild-type (WT) A431 cells compared to cells with a knockout of CD109. Expression levels are normalized to a housekeeping gene (18S) and presented as fold change relative to WT. B) Similar to Panel A, this bar graph depicts the IL6Rα, NRF2, HO1 and SOD1, SOD2 mRNA expression in control (EVA431) cells versus cells engineered to overexpress CD109 [CD109OA431]. Fold change with respect to wtA431 or EVA431cells was calculated using the delta cT method (2−ΔCT). For qPCR, error bars represent ± SEM of three independent experiments performed in triplicates.

**Supplementary Figure 11:** **CD109 is a critical mediator of the interaction between CD109, IL6Rα and GP130 complex.** (A-D) Co-immunoprecipitation (Co-IP) analysis demonstrating the physical interaction between CD109 and IL6Rα or CD109 and GP130 in control (non-transfected), cells transfected with scrambled siRNA and cells transfected with either GP130 siRNA (A-C) or IL6Rα siRNA (B-D) in A431 or SCC9 cells. Western blot bands showing CD109, GP130 and IL6Rα expression levels in A431 and SCC9 cells. A housekeeping protein (β-Actin) is included as a loading control to ensure equal protein loading across samples.

**Supplementary Table 2. Spearman and Pearson correlation between NRF2 target genes and CD109**.

| **CD109** | **Spearman (r)** | **P value** | **Pearson (r)** | **P value** |
| --- | --- | --- | --- | --- |
| AKR1C1 | 0.13 | 5.298e^-3^ | 0.13 | 3.643e^-3^ |
| AKR1C2 | 0.14 | 2.749e^-3^ | 0.17 | 2.036e^-4^ |
| GCLM | 0.2 | 1.203e^-5^ | 0.21 | 1.690e^-6^ |
| AKR1C3 | 0.11 | 0.0144 | 0.13 | 3.989e^-3^ |
| GCLC | 0.19 | 3.905e^-5^ | 0.16 | 2.556e^-4^ |
| TXNRD1 | 0.19 | 1.701e^-5^ | 0.19 | 2.117e^-5^ |
| UCHL1 | 0.13 | 4.413e^-3^ | 0.07 | 0.113 |
| AKR1B10 | 0.12 | 8.477e^-3^ | 0.22 | 5.23e^-7^ |
| NTRK2 | 0.14 | 2.360e^-3^ | 0.12 | 9.761e^-3^ |
| SLC7A11 | 0.19 | 2.799e^-5^ | 0.22 | 1.37e^-6^ |
| SRXN1 | 0.14 | 1.861e^-3^ | 0.18 | 8.477e^-5^ |

List of 11 upregulated NRF2 genes identified in HNSCC. Expression correlations of NRF2 and CD109 in TCGA Head and Neck Squamous cell cancer.

**AKR1C1, AKR1C2, AKR1C3, AKR1B10: Aldo-keto reductase family member C1, C2, C3, B10**

**GCLM: Glutamate-cysteine ligase modifier subunit**

**GCLC: Glutamate-cysteine ligase**

**TXNRD1: Thioredoxin reductase-1**

**UCHL1: Ubiquitin C-terminal hydrolase L1**

**NTRK2: Neurotrophic receptor tyrosine kinase 2**

**SLC7A11: Solute carrier family 7 member 11**

**SRXN1: Sulfiredoxin**

**Primers used for qPCR**

| **Primer name** | **Sequence** |
| --- | --- |
| *18S*_ Sense | 5’AACCCGTTGAACCCCATT3’ |
| *18S_* Antisense | 5’CCATCCAATCGGTAGTAGCG3’ |
| *CD109*_ Sense | 5’GCCCGGAGGAAATGTGACTA3’ |
| *CD109*_ Antisense | 5’ATCCTGGGTACGTCCGGTTA3’ |
| *IL6R*_ Sense | 5’GAGTCATGTGCGAGTGGGAA3’ |
| *IL6R*_ Antisense | 5’CCGGCTCTCTACACACACTG3’ |
| *NRF2* _Sense | 5’AGGTTGCCCACATTCCCAAA3’ |
| *NRF2*_ Antisense | 5’AGTGACTGAAACGTAGCCGA3’ |
| *HO1*_ Sense | 5’AAACTTCAGAGGGGGCGAAG3’ |
| *HO1*_ Antisense | 5’AACCCGTTGAACCCCATT3’ |
| *SOD1*_ Sense | 5’ACAAAGATGGTGTGGCCGAT3’ |
| *SOD1* _Antisense | 5’AACGACTTCCAGCGTTTCCT3’ |
| *SOD2*_ Sense | 5’ GCACTAGCAGCATGTTGAGC3’ |
| *SOD2*_Antisense | 5’TTGATGTGAGGTTCCAGGGC3’ |
