# Supplementary material for: IL-6-mediated tumorigenicity and antioxidant state in squamous cell carcinoma cells are driven by CD109 via stabilization of IL-6 receptor-alpha and activation of STAT3/NRF2 pathway
Source: Exp Hematol Oncol. 2025 May 2;14:64. doi: 10.1186/s40164-025-00630-x (PMC12046912; doi:10.1186/s40164-025-00630-x)
Supplement: Supplementary file 2 — Supplementary Material 2. [file 40164_2025_630_MOESM2_ESM.doc]

**Supplementary Table1: Clinical Data of oral SCC patients who consented and generously provided the samples used for immunohistochemical analysis**.

*This table provides a detailed snapshot of the clinical characteristics and outcomes of the 15 oral SCC patient cohort included in the current study. Patient numbers*

*correspond to tumor and normal tissue numbers in Fig 8A and Supplementary Fig 8.*

Smoking history CURRENT: Patient has smoked cigarettes within 1 year of their diagnosis PREVIOUS: Patient quit smoking cigarettes at least 1 year prior to diagnosis NEVER: Patient has never smoked cigarettes UKNOWN.

Pack-years - 1 pack = 20 cigarettes - Eg. 1 pack-year is equal to smoking 1 pack per day for 1 year.

Alcohol Never: Never >2 drinks per day for >2 weeks Current: >2 alcoholic drinks per day within the last 2 weeks Previous: Currently drink less than above, but at some point in their lives drinking >2 alcoholic drinks per day for >2 weeks
