# Supplementary material for: IL-6-mediated tumorigenicity and antioxidant state in squamous cell carcinoma cells are driven by CD109 via stabilization of IL-6 receptor-alpha and activation of STAT3/NRF2 pathway
Source: Exp Hematol Oncol. 2025 May 2;14:64. doi: 10.1186/s40164-025-00630-x (PMC12046912; doi:10.1186/s40164-025-00630-x)
Supplement: Supplementary file 3 — Supplementary Material 3. [file 40164_2025_630_MOESM3_ESM.docx]

| **CD109** | **Spearman (r)** | **P value** | **Pearson (r)** | **P value** |
| --- | --- | --- | --- | --- |
| AKR1C1 | 0.13 | 5.298e^-3^ | 0.13 | 3.643e^-3^ |
| AKR1C2 | 0.14 | 2.749e^-3^ | 0.17 | 2.036e^-4^ |
| GCLM | 0.2 | 1.203e^-5^ | 0.21 | 1.690e^-6^ |
| AKR1C3 | 0.11 | 0.0144 | 0.13 | 3.989e^-3^ |
| GCLC | 0.19 | 3.905e^-5^ | 0.16 | 2.556e^-4^ |
| TXNRD1 | 0.19 | 1.701e^-5^ | 0.19 | 2.117e^-5^ |
| UCHL1 | 0.13 | 4.413e^-3^ | 0.07 | 0.113 |
| AKR1B10 | 0.12 | 8.477e^-3^ | 0.22 | 5.23e^-7^ |
| NTRK2 | 0.14 | 2.360e^-3^ | 0.12 | 9.761e^-3^ |
| SLC7A11 | 0.19 | 2.799e^-5^ | 0.22 | 1.37e^-6^ |
| SRXN1 | 0.14 | 1.861e^-3^ | 0.18 | 8.477e^-5^ |

**Supplementary Table 2. Spearman and Pearson correlation between NRF2 target genes and CD109**.

List of 11 upregulated NRF2 genes identified in HNSCC. Expression correlations of NRF2 and CD109 in TCGA Head and Neck Squamous cell cancer.
